# ISUOG Consensus Statement on sonographic assessment of the endometrium: how to perform a gynecological ultrasound scan and report the findings

**DOI:** 10.1002/uog.70163

**Published:** 2026-01-16

**Authors:** T. Van den Bosch, R. Heremans, C. Landolfo, E. Epstein, F. P. G. Leone, T. Bourne, D. Timmerman, Collaborators, J. Preisler, O. Rotenberg, H. Marret, G. Condous, J. L. Alcázar, D. Fischerová, U. Metzger, C. Van Pachterbeke, D. Franchi, M. Nisolle, J. Huirne, C. Fotopoulou, S. Goldstein

**Affiliations:** ^1^ Department of Obstetrics and Gynecology University Hospitals Leuven Leuven Belgium; ^2^ Department of Development and Regeneration KU Leuven Leuven Belgium; ^3^ Department of Obstetrics and Gynecology Ziekenhuis Oost‐Limburg ZOL Maas en Kempen Maaseik Belgium; ^4^ Department of Obstetrics and Gynaecology, Queen Charlotte's and Chelsea Hospital Imperial College London London UK; ^5^ Department of Clinical Science and Education, Karolinska Institutet, and Department of Obstetrics and Gynecology Södersjukhuset Stockholm Sweden; ^6^ Department of Obstetrics and Gynecology Clinical Sciences Institute Luigi Sacco Milan Italy

## Clinical Standards Committee

The International Society of Ultrasound in Obstetrics and Gynecology (ISUOG) is a scientific organization that encourages sound clinical practice and high‐quality teaching and research related to diagnostic imaging in women's healthcare. The ISUOG Clinical Standards Committee (CSC) has the remit to develop Practice Guidelines and Consensus Statements as educational recommendations that provide healthcare practitioners with a consensus‐based approach, from experts, for diagnostic imaging. They are intended to reflect what is considered by ISUOG to be the best practice at the time at which they are issued. Although ISUOG has made every effort to ensure that Guidelines and Consensus Statements are accurate when issued, neither the Society nor any of its employees or members accepts any liability for the consequences of any inaccurate or misleading data, opinions or statements issued by the CSC. The ISUOG CSC documents are not intended to establish a legal standard of care because interpretation of the evidence that underpins them may be influenced by individual circumstances, local protocol and available resources. Approved Guidelines and Consensus Statements can be distributed freely with the permission of ISUOG (info@isuog.org).

## INTRODUCTION

The aim of ultrasonography of the endometrial cavity is to evaluate the physiological and pathological changes of the endometrium and detect intracavitary lesions. In 2010, the International Endometrial Tumor Analysis (IETA) consortium^1^ proposed a structured lexicon to standardize the description of the sonographic features of the endometrium and intrauterine lesions. In recent years, several IETA studies^2^, ^3^, ^4^, ^5^ have applied this lexicon to report the typical sonographic features of normal and abnormal endometrial changes and intracavitary lesions.

The initiative for this Consensus Statement came from the ISUOG CSC. Applying the IETA lexicon, we explain how to perform a gynecological ultrasound scan, focusing on endometrial evaluation, and discuss the reporting of sonographic endometrial findings, focusing on clinical relevance for diagnosis and management. This Consensus Statement will be of interest not only to gynecologists with a special interest in ultrasonography, but also to general gynecologists, those performing hysteroscopy, gynecological oncologists and fertility specialists. Pregnancy‐related findings, Cesarean scar defects, congenital uterine anomalies and cancer staging are beyond the scope of this Consensus Statement.

The contributors to this Consensus Statement are all experts in their fields and have expressed their opinions based on the available literature and evidence. Any clinician applying this Consensus Statement is expected to use their independent medical judgment in the context of the individual clinical circumstances to determine patient care.

## METHODS

Twenty experts in the field of gynecological ultrasonography, hysteroscopy and oncology were invited to participate based on their expertise and geographic spread. An online poll was created to assess agreement among all coauthors on the relevant statements for the topics under discussion (Table 1). Statements that were fully or partially agreed upon by ≥ 90% of the voters remained unaltered. Nine statements did not meet this threshold and were reformulated and circulated for a second poll. Both polls were completed by 19 of the 20 participants.

**Table 1:** Summary of online poll sent out during development of this Consensus Statement, to assess agreement among participants regarding the topics under discussion

Statements	Agreement (*n*/*N* (%))					
	Full	Partial	Uncertain	Disagree	Grade of Rec.	Level of Evidence
*Basic recommendations*						
1. If not contraindicated, and after informed consent has been obtained, transvaginal ultrasonography is the method of choice to evaluate the endometrium^1^.	19/19 (100)	0/19 (0)	0/19 (0)	0/19 (0)	D	4
2. Before menopause, in women with a spontaneous cycle, the optimal timing of an ultrasound examination to detect endometrial lesions is in the proliferative phase of the cycle, after the end of menstruation^1^.	18/19 (94.7)	1/19 (5.3)	0/19 (0)	0/19 (0)	D	2–
3. In postmenopausal women on sequential hormone replacement therapy, the optimal timing of an ultrasound examination to detect endometrial lesions is in the estrogen‐only phase of the cycle, after the end of withdrawal bleeding^1^.	19/19 (100)	0/19 (0)	0/19 (0)	0/19 (0)	C	2+
4. In pre‐ and perimenopausal women, repeating the ultrasound scan after the next menses or hormonally induced withdrawal bleeding may optimize the ultrasound image^1^.	18/19 (94.7)	1/19 (5.3)	0/19 (0)	0/19 (0)	GPP	4
5. *In any patient presenting with abnormal uterine bleeding, a non‐endometrial etiology should be considered (especially in the absence of obvious endometrial pathology): e.g. pathology of the cervix, the ovaries, the bladder and the rectum, as well as clotting defects and hormonal factors^1^, ^62^.	18/19 (94.7)	1/19 (5.3)	0/19 (0)	0/19 (0)	GPP	4
6. *During transvaginal ultrasonography, the presence of a chaperone might be advisable in adolescents, patients who have had previous negative experience with gynecological (sonographic) examinations, anxious patients, elderly women and women who are unable to give informed consent^6^, ^7^.	16/19 (84.2)	3/19 (15.8)	0/19 (0)	0/19 (0)	GPP	4
7. *Transvaginal ultrasonography is contraindicated in women who are unable to give informed consent, when there is vaginal stenosis (e.g. secondary to atrophy, radiotherapy), if the patient is *virgo intacta* or in the presence of other contraindications for introducing a vaginal probe (e.g. risk of heavy bleeding from an exophytic cervical cancer or after recent vaginal surgery)^7^.	14/19 (73.7)	5/19 (26.3)	0/19 (0)	0/19 (0)	GPP	4
8. *A transrectal scan should be used with caution, tailored to the individual, and is not advised in women with impaired mental health and in case of anal pathology (e.g. anal fissure)^8^.	16/19 (84.2)	3/19 (15.8)	0/19 (0)	0/19 (0)	D	2+
*Reporting endometrial thickness and sonographic features*						
9. Endometrial thickness is measured in the sagittal plane (where it appears thickest) and recorded in millimeters, conforming to the 2010 IETA Consensus Opinion^1^. If the endometrium cannot be visualized over the entire cavity, it should be reported as ‘non‐measurable’^1^.	18/19 (94.7)	1/19 (5.3)	0/19 (0)	0/19 (0)	D	4
10. The sonographic features of the endometrium should be described according to the 2010 IETA Consensus Opinion^1^.	18/19 (94.7)	1/19 (5.3)	0/19 (0)	0/19 (0)	D	4
11. It is advisable to include color/power Doppler in the routine sonographic examination of the endometrium^1^.	13/19 (68.4)	6/19 (31.6)	0/19 (0)	0/19 (0)	B	2++
12. Although endometrial thickness and sonographic features may be indicative of a specific pathology, ultrasonography should not replace histology.	17/19 (89.5)	2/19 (10.5)	0/19 (0)	0/19 (0)	B	2++
*Sonohysterography*						
13. If the endometrium cannot be identified in its entirety, or in case of doubt about the presence of an intracavitary lesion, sonohysterography using sterile saline or gel should be considered^1^.	18/19 (94.7)	1/19 (5.3)	0/19 (0)	0/19 (0)	C	2+
14. *If sonohysterography is used in cases at high risk for malignancy, it is good clinical practice to use as little fluid and as low instillation pressure as possible. In these cases, the use of gel instead of saline might be considered^39^.	14/19 (73.7)	5/19 (26.3)	0/19 (0)	0/19 (0)	GPP	4
15. *Prophylactic antibiotics in sonohysterography may be considered in a high‐risk population.	13/19 (68.4)	5/19 (26.3)	0/19 (0)	1/19 (5.3)	GPP	4
16. Sonohysterography is contraindicated in the presence of a hydrosalpinx, frozen pelvis, suspicion of cervicitis or pelvic infection and if pregnancy cannot be ruled out^43^.	18/19 (94.7)	1/19 (5.3)	0/19 (0)	0/19 (0)	GPP	4
*3D ultrasound*						
17. 3D sonography is not mandatory in the diagnosis of endometrial lesions but may help in the preoperative mapping of intracavitary lesions (e.g. fibroids, polyps)^54^.	17/19 (89.5)	2/19 (10.5)	0/19 (0)	0/19 (0)	C	2+
*Management*						
18. Management depends not only on the endometrial sonographic features, but also on other clinical factors (e.g. patient age or comorbidity), and requires a well‐informed and shared decision with the patient^86^.	19/19 (100)	0/19 (0)	0/19 (0)	0/19 (0)	GPP	4
19. Further testing is not necessary in a woman presenting with postmenopausal bleeding, provided that: (1) the endometrium is clearly visible over its entirety, (2) the endometrial thickness does not exceed 4 mm, (3) there is an absence of internal endometrial vascularity and (4) cervical pathology and other gynecological causes have been ruled out.	16/19 (84.2)	2/19 (10.5)	0/19 (0)	1/19 (5.3)	C	2+
*Asymptomatic women*						
20. Routine screening of the endometrium using ultrasound is not indicated in asymptomatic (i.e. no abnormal uterine bleeding), low‐risk, postmenopausal women^74^.	18/19 (94.7)	1/19 (5.3)	0/19 (0)	0/19 (0)	C	2+
21. Routine screening of the endometrium using ultrasound is not indicated in asymptomatic (i.e. no abnormal uterine bleeding) women on tamoxifen^71^, ^73^.	16/19 (84.2)	3/19 (15.8)	0/19 (0)	0/19 (0)	C	2+
22. *In a woman without abnormal uterine bleeding, the incidental finding of two or more ‘alarm features’ (alarm features include: (1) a thickened endometrium > 4 mm, (2) non‐uniform endometrium with irregular outline, (3) multiple vessels with or without branching), indicates that further testing should be considered.	14/19 (73.7)	4/19 (21.1)	0/19 (0)	1/19 (5.3)	GPP	4
*Endometrial lesion*						
23. In women presenting with a focal endometrial lesion and abnormal uterine bleeding, hysteroscopic removal should be proposed.	18/19 (94.7)	1/19 (5.3)	0/19 (0)	0/19 (0)	C	2+
24. In the absence of abnormal bleeding, the presence of multiple vessels with an irregular branching pattern as well as the observation of an irregular endometrial echogenicity or outline of the intracavitary lesion indicates that endometrial sampling should be considered.	17/19 (89.5)	2/19 (10.5)	0/19 (0)	0/19 (0)	GPP	4
*Endometrial sampling*						
25. *In case of a focal lesion found at sonohysterography, hysteroscopy is the preferred sampling technique, while in case of a globally thickened endometrium, blind sampling techniques may be adequate^88^.	18/19 (94.7)	1/19 (5.3)	0/19 (0)	0/19 (0)	C	2 +
26. *If endometrial malignancy is suspected based on sonographic features, office endometrial sampling is indicated^89^. The sample should be processed with priority. If the biopsy result is inconclusive or shows only benign findings, hysteroscopy is recommended.	17/19 (89.5)	2/19 (10.5)	0/19 (0)	0/19 (0)	D	2+
27. During endometrial sampling, the tissue yield should be assessed and compared to the sonographic features (in case of minimal tissue yield in a woman with a thickened endometrium at ultrasound scan, a lesion has most probably been missed, and the results of the histology will most probably not be representative)^92^, ^93^, ^94^, ^95^.	18/19 (94.7)	1/19 (5.3)	0/19 (0)	0/19 (0)	C	2+

Details of Grades of Recommendation (Rec.) and Levels of Evidence used in International Society of Ultrasound in Obstetrics and Gynecology Guidelines and Consensus Statements are given in Appendix 1.

* Statements that did not meet the threshold of full or partial agreement by ≥ 90% of voters; these were reformulated and sent for a second appraisal, and final statements and agreement are presented. 3D, three‐dimensional; GPP, good practice point; IETA, International Endometrial Tumor Analysis.

## HOW TO PERFORM A GYNECOLOGICAL ULTRASOUND SCAN, WITH FOCUS ON ENDOMETRIAL EVALUATION

### History

For the optimal interpretation of an ultrasound examination, the referring clinician should state clearly the reason(s) for referral and share the relevant clinical information (e.g. patient's age, height, weight, body mass index, medication, comorbidity, previous sonographic findings, latex allergy) and relevant gynecological history, including last menstrual period, age at menopause, hormonal/non‐hormonal contraceptive use, hormonal replacement therapy (HRT), gravidity, parity, previous Cesarean section, previous miscarriage or previous surgery.

### Setting

Gynecological ultrasonography should be performed in a reassuring environment. The absence of latex allergy needs to be confirmed when working with latex probe covers. It is advised to have latex‐free alternatives (e.g. nitrile gloves). Fully informing the patient and obtaining their consent before proceeding with the examination are essential. The presence of a chaperone might be particularly advisable in adolescents, patients who have had previous negative experience with gynecological (sonographic) examinations, anxious patients or elderly women^6^. The presence of a chaperone is, whenever possible, offered in the UK and in many other countries in all cases requiring intimate examination. This is mandatory in the UK for children, adolescents and women who are unable to give informed consent^6^, ^7^.

### Inspection

Prior to vaginal ultrasonography, it is advised to exclude any vulvar or perineal lesions by external examination. Scanning may cause discomfort, thus it is important to communicate with the patient, to reassure them and ask them to tell the examiner whether they experience any anxiety, pain or discomfort^8^, ^9^.

### Timing of the scan

**Figure 1:**
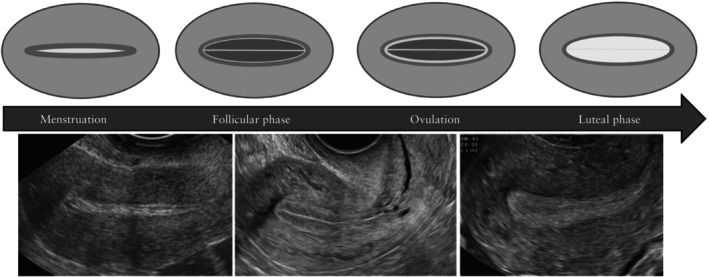
Diagrams and ultrasound images illustrating the physiological endometrial appearance and changes throughout the menstrual cycle.

Before menopause, an ultrasound examination to detect endometrial lesions should preferably be performed in the early proliferative phase of the menstrual cycle (i.e. after cessation of menstrual bleeding) or after withdrawal bleeding in women taking combined hormonal contraception; in postmenopausal women on cyclic HRT, it should be performed in the estrogen‐only phase of the cycle, after the end of withdrawal bleeding, or 5–10 days after the last progestin tablet, depending on the HRT scheme. In the first phase of the menstrual cycle, the endometrium typically has a uniform, hypoechogenic appearance at ultrasound, with a regular, linear midline (i.e. a three‐layer pattern). A focal intracavitary lesion, such as an endometrial polyp, will distort the endometrial midline and will be easily visible against the hypoechogenic background. During menstruation, the presence of intracavitary blood (clots) may obscure the view and impede proper endometrial evaluation. In the secretory phase of the cycle, the endometrium becomes more echogenic and, at the end of the cycle, may even develop a more polypoid growth pattern (Figure 1). This may mask or mimic an intracavitary lesion. If imaging is suboptimal, especially in those presenting late in their menstrual cycle, it may be advisable to ask the patient to come back for a second ultrasound evaluation after the next bleed.

### Scanning technique

Ultrasonography of the endometrium may be performed using a transvaginal, transabdominal or, less commonly, transperineal or transrectal approach. According to some local protocols, it is customary to perform both transabdominal and transvaginal ultrasonography in every patient.

#### Transvaginal ultrasonography

In most adult women, the endometrium and uterine cavity may be scanned transvaginally^7^. Transvaginal scanning is the preferred approach, but is contraindicated in women who are unable to give informed consent, when there is vaginal stenosis (e.g. secondary to atrophy, radiotherapy), if the patient is *virgo intacta* or in the presence of other contraindications for introducing a vaginal probe (e.g. risk of heavy bleeding from an exophytic cervical cancer or after recent vaginal surgery). The management of adolescents should be individualized.

In most cases, the patient should be asked to empty her bladder before the examination. The patient is examined in the lithotomy position. Endocavitary ultrasound transducers are considered semicritical medical devices owing to the high risk of potential contamination^10^. They therefore require an appropriate probe cover and should undergo high‐level disinfection after use. A probe cover, filled with gel, is placed on the probe and sufficient gel is applied to the cover before the probe is introduced gently into the vagina. In some countries the use of individual gel sachets is mandatory in view of the risk of infection.

In some cases (e.g. cases of postmenopausal bleeding or when there is suspicion of endometriosis), it is advised to evaluate the bladder wall to exclude a bladder lesion (e.g. polyps, primary transitional cell carcinoma or metastatic disease^11^) or deep endometriosis^12^. In these circumstances, a minimal level of bladder filling is needed. Therefore, if the bladder is empty, the ultrasound examination may be repeated after 30 min to allow some bladder filling.

For optimal interobserver agreement, it is advisable to keep image orientation (cranial, caudal, left, right) consistent within the same unit/department, and to specify the orientation if deviating from the unit's protocol.

Ultrasound scanning is a dynamic examination^13^. For example, gentle pressure on the uterus may allow differentiation between a blood clot and a sessile endometrial polyp.

Selective site‐specific tenderness when applying pressure on (part of) the uterus may suggest endometritis or adenomyosis^12^, ^14^.

#### Transabdominal ultrasonography

A transabdominal scan may be preferred when the uterus has an axial position (i.e. when the long axis of the endometrium is continuous with and parallel to the long axis of the endocervical canal), in the presence of large fibroids or a globally enlarged uterus (in which case, having an empty bladder may be advisable) or when a transvaginal scan is considered inappropriate^8^.

In most cases, before carrying out a transabdominal scan, the patient should be instructed to have a full bladder^15^. A (partially) full bladder pushes the bowel loops upwards, which creates an acoustic window, enhancing the ultrasound signal. However, an overfilled bladder causes patient discomfort and increases the focal distance between the ultrasound probe and the target organ, decreasing image quality.

Applying gentle pressure with the probe optimizes skin contact, decreases the focal distance and may move overlying structures (e.g. bowel loops) aside. Slight flexion of the patient's legs relaxes the abdominal wall muscles, maximizing contact between the probe and the abdomen.

In patients with morbid obesity or extensive lower abdominal scarring, transabdominal ultrasonography may result in poor image quality. This should be mentioned in the scan report. In such cases, a vaginal probe may be used, placing it into the umbilicus, where the abdominal wall is usually at its thinnest. In patients with morbid obesity, the umbilicus is often located more caudally, which may also result in improved imaging. Placing the transabdominal transducer cranially to the symphysis pubis, immediately below the abdominal panniculus, may also be helpful.

#### Transperineal ultrasonography

A transperineal approach can be considered to assess perineal tissues, the vagina and the uterine cervix^16^, ^17^. In normal circumstances, satisfactory views of the endometrium are rarely obtained owing to suboptimal focal depth and angulation of the uterus. However, in the presence of significant hematometra or uterine prolapse, the endometrium of the lower uterine segment or even midcavity can be assessed adequately.

#### Transrectal ultrasonography

Transrectal scanning yields an image quality similar to that of the transvaginal route^18^. Indications for transrectal scanning are vaginal stenosis or other contraindications for introducing a vaginal probe.

A transrectal ultrasound scan may be considered to be intrusive. Comprehensive explanation and informed consent are mandatory^8^. The management of adolescents should be tailored to the individual. If an adolescent has not had previous sexual intercourse, transrectal ultrasonography may be performed, provided the patient is well informed and accompanied and the examination is carried out in a welcoming and relaxed environment. Transrectal assessment should be avoided in women who are unable to understand or consent to the procedure, for example women with impaired mental health, and in the presence of anal pathology (e.g. anal fissure). In these patients, alternative imaging modalities (e.g. transabdominal ultrasound or magnetic resonance imaging) can be offered.

A vaginal probe can be used for transrectal ultrasound. Before inserting the probe, a lubricant is applied to the anal canal. A digital anal examination is performed first, to indicate the direction of the anal canal and to relax the anal sphincter. The finger is then withdrawn and the probe is pushed gently through the anal canal immediately afterwards. During insertion, the patient is asked to ‘push as if passing stools’ to relax the anal sphincter. Once the tip of the probe has passed through the anal sphincter, the examination is usually well tolerated.

### Optimizing image quality

Every assessment of the uterus should start with identification of the bladder and the cervix^1^. The position of the uterus is noted and measurements taken. The uterus is scanned in the sagittal plane from *cornu* to *cornu* and in the transverse (oblique) plane from the cervix to the fundus. Having established an overview of the whole uterus, the image is magnified to contain only the uterine corpus. The magnification should be as large as possible, focusing on the area of interest. In general, the endometrium is easily visualized. The probe frequency should be adapted to the focal depth: for structures near to the probe, a higher frequency is chosen, while a lower frequency will optimize the visualization of deeper tissues.

Suboptimal imaging may arise from variations in uterine position (particularly when axial) or with uterine rotation (e.g. owing to endometriosis or previous surgery‐related adhesions). Further problems may be encountered when the cavity is distorted by coexisting benign pathology, such as adenomyosis or fibroids. Endometrial cancer may also distort the endometrial–myometrial interface^3^, ^19^, ^20^, ^21^. Malignancy should therefore be considered as a possible explanation when the endometrium is poorly defined.

When the endometrium is difficult to visualize, it may be helpful to trace it from the endocervical canal. If possible, the angle of insonation between the endometrium and the ultrasound beam should be 90° to optimize image quality^1^.

If the endometrium cannot be seen, saline or gel instillation often provides substantial additional information on the endometrium and the uterine cavity^1^, ^22^, ^23^, ^24^. For an axial uterus, a transabdominal scan may provide improved images if the endometrium cannot be visualized transvaginally.

### Color and power Doppler

In many centers, color or power Doppler is integrated into the routine ultrasound examination^1^. The use of color or power Doppler is of added value for the detection of intracavitary lesions, such as endometrial polyps, intracavitary fibroids, endometrial malignancy or retained pregnancy tissue^19^, ^25^, ^26^, ^27^.

Color and power Doppler reflect the amount of blood flow present. The color signal in the endometrium may be scored using a subjective semiquantitative assessment: a color score of 1 is assigned when no color‐flow signals can be identified, a score of 2 when only minimal color can be detected, a score of 3 when moderate color is present and a score of 4 when abundant color is detected^1^. The optimal pulse‐repetition frequency for the detection of vessels within the uterus is between 0.3 and 0.6 kHz, which corresponds to a velocity scale between 3.0 and 6.0 cm/s. Endometrial blood flow is best appreciated when the direction of flow is parallel to the ultrasound beam.

When evaluating vascular patterns of the endometrium, the region of interest should include the myometrium adjacent to the endometrium, to allow accurate assessment of flow across the endometrial–myometrial junction.

**Figure 2:**
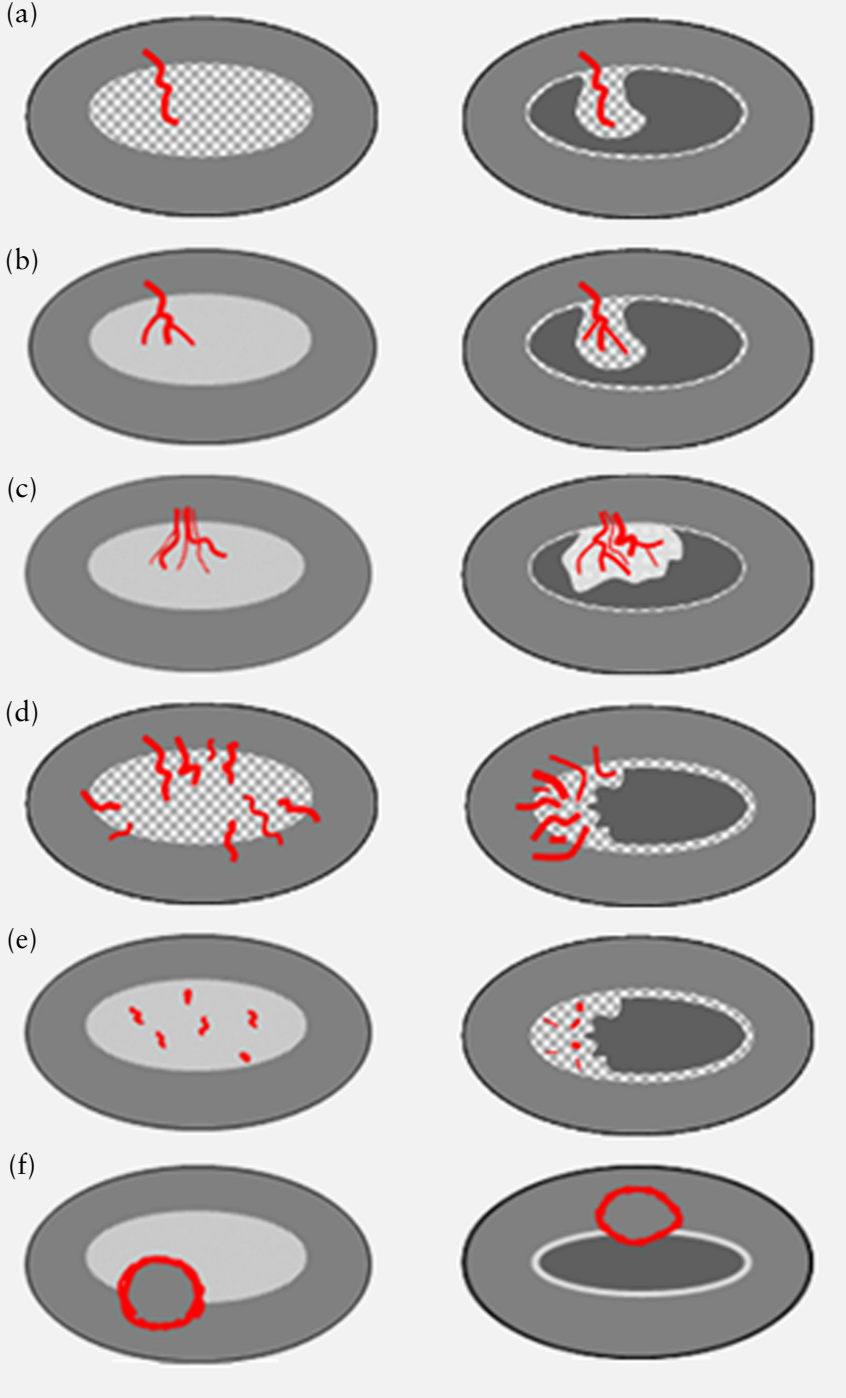
Diagrams showing different endometrial vascular patterns observed with power/color Doppler: unenhanced ultrasonographic (left) and sonohysterographic (right) imaging. (a) Single dominant vessel without branching. (b) Single dominant vessel with branching. (c) Multiple vessels with focal origin (two or more vessels appear to share a common stem). (d) Multiple vessels with multifocal origin at the endometrial–myometrial junction. (e) Scattered vessels (dispersed color signals within the endometrium but without visible origin at the endometrial– myometrial junction). (f) Circular flow. Adapted from Leone *et al*.^1^.

The vascular pattern within the endometrium is reported with reference to the presence of a ‘dominant vessel’, the number of vessels, the presence of branching, the regularity of branching and whether blood vessels flowing into the endometrium have a focal or multifocal origin^1^ (Figure 2).

Dominant vessels are defined as one or more distinct (arterial and/or venous) vessels passing the endometrial–myometrial junction^1^. The dominant vessel may show branching within the endometrium, which is described as either regular/orderly or irregular/disorderly/chaotic. Dominant vessels may present as a single vessel (pedicle artery sign), with or without branching^26^. Multiple dominant vessels may have a focal origin at the endometrial–myometrial junction or a multifocal origin. Other vascular patterns within the endometrium include scattered vessels (dispersed color signals within the endometrium but without visible origin at the endometrial–myometrial junction) and circular flow^1^.

#### Pitfalls

Errors in image interpretation, such as an apparent absence of flow, may be caused by a transient myometrial contraction^28^, ^29^, ^30^, ^31^. Furthermore, flow may not be apparent at times because the vessels are oriented perpendicular to the ultrasound beam. Excessive pressure with the probe or excessive distance from the endometrium may also impair the detection of blood flow.

### Sonohysterography

Sonohysterography involves the instillation of sterile fluid into the uterine cavity to enhance visualization on ultrasound^1^, ^22^. The anechoic fluid acts as a negative contrast agent. Either saline (saline contrast sonohysterography) or gel (gel instillation sonography) may be used.

Sonohysterography is indicated if the endometrium cannot be visualized in its entirety or if an intracavitary lesion is suspected^1^. Sonohysterography and hysteroscopy have similar reported diagnostic accuracy for the visualization of intracavitary lesions^32^, ^33^, ^34^.

**Figure 3:**
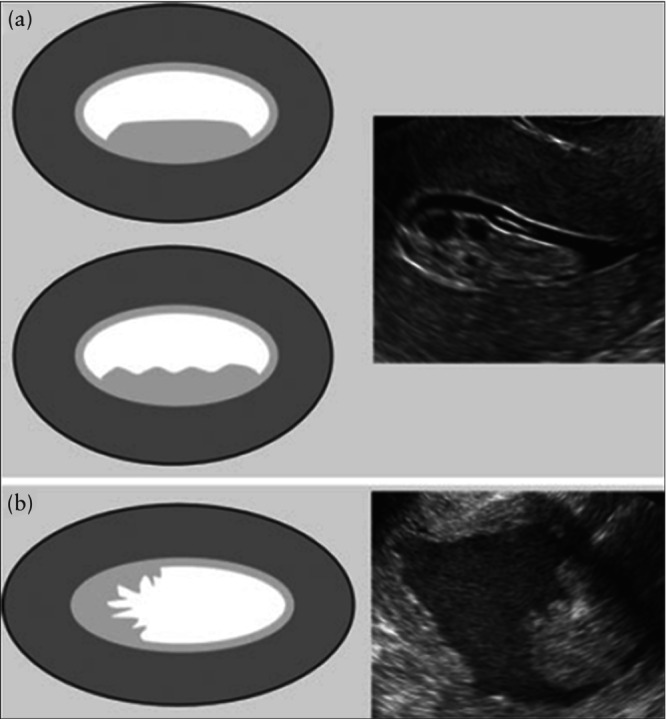
Diagrams and ultrasound images showing endometrial or lesional outline at sonohysterography or when there is pre‐existing fluid in the uterine cavity: (a) smooth (upper diagram) or polypoid (lower diagram) outline; and (b) ‘spiky’/irregular outline. Reproduced with permission from Leone *et al*.^1^.

The endometrial outline is defined as smooth if the endometrial surface facing the uterine cavity appears regular, and as having endometrial folds, or as ‘polypoid’, if there are deep indentations^1^ (Figure 3a). The endometrium is described as ‘irregular’ if the surface facing the uterine cavity is cauliflower‐like or sharply toothed (‘spiky’) (Figure 3b). In the presence of a spiky intracavitary lesion, the differential diagnosis must be made between a (potentially malignant) endometrial lesion and a blood clot. Using power Doppler, a blood clot will appear avascular. Unlike an endometrial lesion, a clot often moves within the cavity when gentle pressure is applied with the ultrasound probe^35^, ^36^.

#### How to perform sonohysterography

Air bubbles are strongly echogenic and impact significantly on image quality^37^. It is essential to avoid the presence of air bubbles within the instilled fluid and to carefully flush all the air present within the syringe and the catheter before inserting the catheter into the endocervical canal^38^.

Different catheters can be used (e.g. neonatal suction catheters, pediatric nasogastric tubes, insemination catheters or dedicated sonohysterography catheters). If the cervical os is stenotic, it can be gently dilated mechanically or distended carefully by applying hydrodilatation.

Once the catheter has been inserted into the cavity, the fluid is injected slowly under direct ultrasound guidance. In most cases, minimal uterine cavity distension using 1–3 mL of fluid is sufficient to allow optimal evaluation of the endometrium and the uterine cavity^39^.

Sonohysterography is usually well tolerated and does not require any pain medication^40^. However, some women may experience lower abdominal cramps during and/or after fluid instillation. This is most likely in women with a history of severe dysmenorrhea or deep dyspareunia. In these patients, in the absence of contraindications, the use of a non‐steroidal anti‐inflammatory drug 30 min before fluid instillation may be considered^38^, ^41^, ^42^.

There is no consensus on the use of prophylactic antibiotics before sonohysterography in asymptomatic women. Most centers do not routinely prescribe prophylactic antibiotics, while some use them in women presenting for fertility investigations. In high‐risk populations (e.g. patients at high risk of pelvic infection or with a pre‐existing cardiac condition) prophylactic antibiotics may be considered.

#### Contraindications

Sonohysterography is contraindicated in the presence of a hydrosalpinx, frozen pelvis or active or previous pelvic infection, on suspicion of cervicitis and if pregnancy cannot be ruled out^43^.

In fertile women, sonohysterography should not be performed in the second half of the menstrual cycle, in order to avoid false‐positive findings due to normal secretory endometrial folds, which may give a polypoid appearance, and to avoid the possibility of an early pregnancy (unless the latter is deemed impossible)^1^.

Similar to hysteroscopy^44^, ^45^, sonohysterography may cause transtubal seeding of (potentially malignant) endometrial cells into the abdominal cavity^46^, ^47^, ^48^. Although its negative impact on prognosis has not been demonstrated, some experts recommend that, in cases considered highly suspicious for cancer, saline infusion should be avoided or, if performed, the pressure and the volume of saline instillation should be kept to a minimum. Owing to its higher viscosity, gel is less likely to flow through the Fallopian tubes^39^. Some authors therefore advocate the use of gel instead of saline to minimize the risk of seeding^39^.

If there is active bleeding in the early postpartum period, saline is preferred to gel infusion. Gel tends to mix with blood and clots, blurring the image, and gel emboli may cause transient hypoxia if there are dilated vessels opening into the uterine cavity and large volumes of gel are instilled^42^, ^49^.

### Three‐dimensional ultrasound imaging

**Figure 4:**
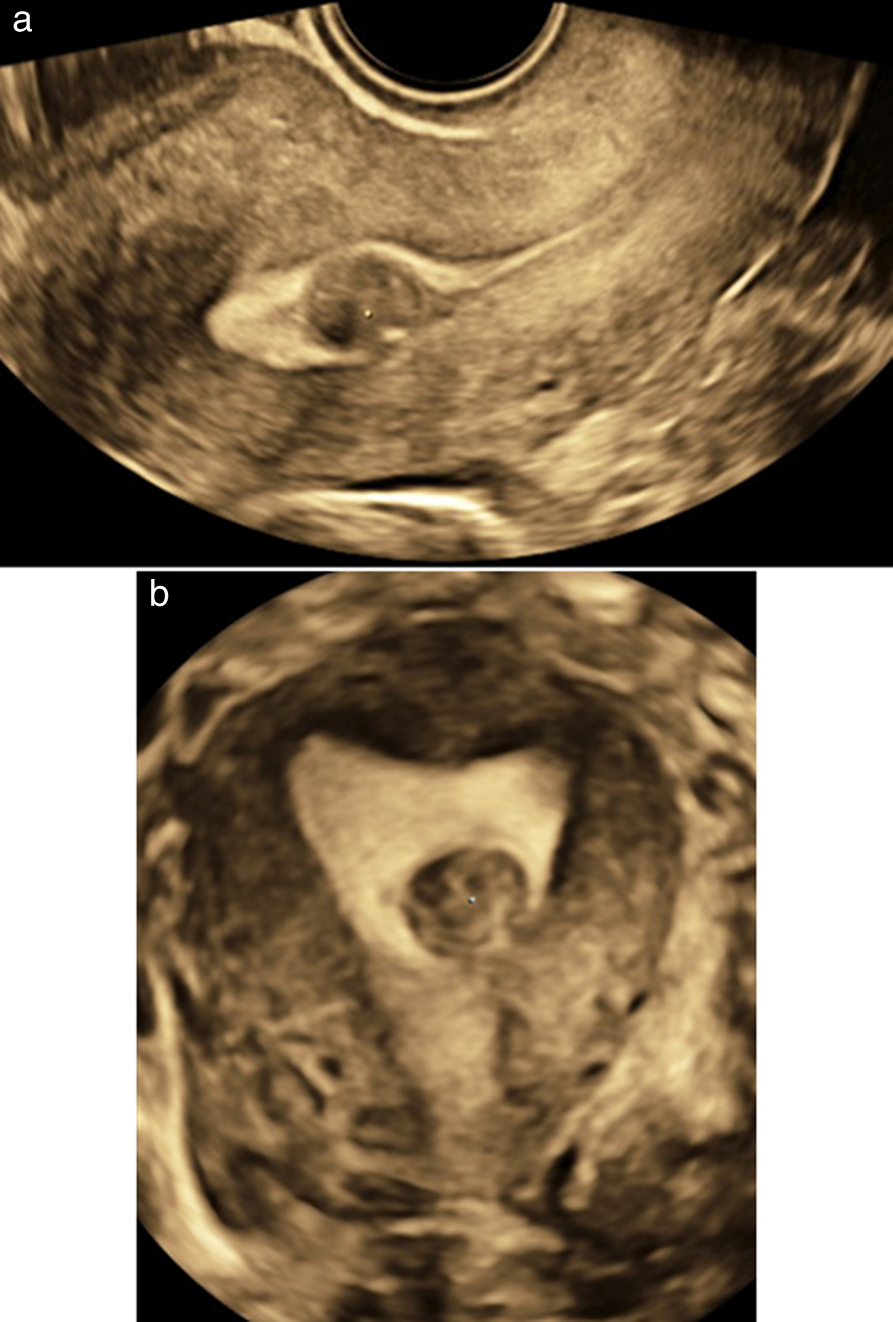
Example of an intracavitary fibroid visualized on three‐dimensional ultrasound, in sagittal (a) and frontal (b) section.

Three‐dimensional (3D) ultrasound imaging is not mandatory for endometrial lesions but is recommended for the evaluation of congenital uterine anomalies^50^, ^51^, ^52^, ^53^, ^54^, and is of added value for the correct preoperative mapping of intracavitary fibroids and polyps (Figure 4), the location of retained pregnancy tissue with enhanced myometrial vascularity, intrauterine contraceptive device/system (IUCD/IUS) localization and the evaluation of Cesarean section scars^1^, ^14^, ^54^, ^55^, ^56^.

A 3D scan may also be of help if the uterus is rotated laterally, preventing two‐dimensional acquisition of a midsagittal section. After 3D volume acquisition, offline rotation within the sectional planes often allows reconstruction of an appropriate midsagittal plane^57^.

In clinical practice, immediate evaluation of the 3D volume during the examination is advisable to assess the image quality and the need for additional volume acquisitions^58^. Providing that the examiner has been properly trained, this may be performed swiftly, avoiding more time‐consuming secondary testing.

### Tips and tricks

#### Endometrium not visible or not measurable

If the endometrium is not clearly visible, the following steps may be attempted to obtain a better angle of insonation and improve image quality:

##### Patient position on the table

The patient may be asked to slide downwards on the examination table, tilting the pelvis and flexing the hips.

##### Bimanual palpation and gentle pressure

Using the transvaginal probe, gentle pressure may be applied to the uterus from the anterior, posterior or lateral vaginal fornix^13^. Using the non‐scanning hand, the examiner may apply some pressure on the abdomen to move the uterus or the overlying organs. In some cases, waiting for some bladder filling or for transient myometrial contractions to set in or subside may improve the angle of insonation.

Gently pushing the transvaginal probe against the uterine corpus, causing the two endometrial surfaces to slide against each other, may aid in detecting the endometrial midline and/or small intracavitary lesions.

##### Transabdominal (or transperineal or transrectal) scan

In case of an anteverted or axial uterus, transabdominal scanning (with the patient's bladder partially filled or empty, according to circumstances) may give better ultrasound images. A full bladder may be ideal in some cases, as it helps to position the endometrium more perpendicular to the ultrasound beam, which is optimal for its evaluation.

##### Fluid instillation

Often, fluid instillation will allow better imaging of the endometrium and the uterine cavity.

##### Repeating the scan

Often, in the (late) secretory phase and during menses, the endometrium is difficult to evaluate accurately. Repeating the examination in the proliferative phase of the cycle, when the endometrium is uniformly hypoechogenic or trilayer, without intracavitary blood or clots, will generally allow proper evaluation.

#### Dealing with blood clots

Blood clots may be difficult to differentiate from endometrial tissue^38^. During fluid instillation, clots may have an irregular outline, mimicking a malignant lesion. The following maneuvers may help to differentiate between a clot and (malignant) endometrial tissue:

##### Color/power Doppler

On color/power Doppler examination a clot is entirely avascular. However, although the presence of vessels is indicative of an endometrial lesion, their absence does not rule one out.

##### Pressure

Applying gentle pressure with the probe may cause a clot to slide over the endometrial wall.

##### Fluid instillation

During fluid instillation a clot often moves and its shape tends to change. The irrigation can also serve to flush debris from the cavity.

##### Pipelle aspiration

If there is still doubt, a Pipelle aspiration can be performed to confirm the presence of a clot, and follow‐up ultrasound can confirm its disappearance post‐aspiration^38^.

#### Subendometrial cysts and adenomyosis

Subendometrial cysts, due to tamoxifen therapy or to adenomyosis, should be distinguished from endometrial cysts (e.g. within an endometrial polyp). If in doubt, fluid instillation may help to make the distinction^38^.

In adenomyosis, involvement of the junctional zone may blur the endometrial–myometrial junction. In some cases, this may preclude measurement of endometrial thickness. This should be reported accordingly^14^.

Junctional‐zone involvement in (severe) adenomyosis may not always be distinguishable from malignant myometrial invasion^1^. Focal increased vascularity increases the probability of malignancy. The possibility of the ‘worst‐case scenario’ should be reported first, followed by the differential diagnosis.

#### Shadowing

Shadowing due to the presence of (calcified) fibroids or intrauterine devices may hamper imaging. In such a case, moving the probe to optimize the angle of insonation may help^1^. The presence of shadowing and its cause should be mentioned in the scan report^14^.

## HOW TO REPORT SONOGRAPHIC ENDOMETRIAL FINDINGS, WITH FOCUS ON CLINICAL RELEVANCE FOR DIAGNOSIS AND MANAGEMENT

### Reporting the ultrasound scan

If the endometrium cannot be seen clearly in its entirety, it should be reported as ‘non‐measurable’ and no attempt should be made to measure it. The proportion of cases in which the endometrium cannot be measured may exceed 10%^59^, especially in older patients^60^.

#### Endometrial thickness

**Figure 5:**
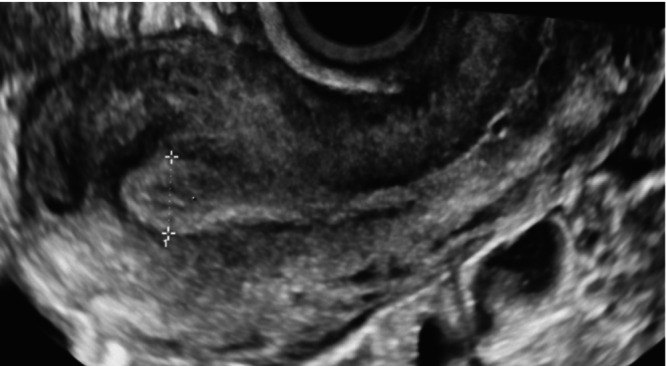
Example of double‐layer measurement of endometrial thickness on ultrasound (sagittal section). Calipers should be placed on the two opposite endometrial–myometrial interfaces in an appropriately magnified image (with the full length of the endometrial cavity visible and the uterus filling more than half of the screen), and the endometrium measured at the point at which it appears to be thickest, measuring perpendicular to the endometrial midline.

Endometrial thickness should be measured at its maximum point in the sagittal plane and include both endometrial layers (double‐layer endometrial thickness)^1^ (Figure 5). The calipers should be placed on the two opposite endometrial–myometrial interfaces in an appropriately magnified image, and the endometrium should be measured at the point at which it appears to be thickest, measuring perpendicular to the endometrial midline. The measurement should be reported in millimeters, rounded up to one decimal point.

When intracavitary fluid is present, the thickness of both single layers should be measured, and their sum recorded. In the presence of an intracavitary lesion (e.g. polyp), the total endometrial thickness should include the diameter of the lesion in the sagittal plane as well as both underlying anterior and posterior layers of the background endometrium. However, if an intracavitary myoma is clearly identified, the myoma should not be included in the measurement of endometrial thickness. Intracavitary lesions should be measured in three perpendicular diameters in millimeters, rounded up to one decimal point. If an intracavitary lesion is present, the total endometrial thickness is less relevant than the measurement and description of the lesion itself.

The echogenicity of the intracavitary fluid should be specified (e.g. anechoic, low level, hemorrhagic).

#### Morphology

An evaluation of endometrial morphology should include assessment of endometrial echogenicity, the endometrial midline and the endometrial–myometrial junction^1^.

##### Echogenicity

The echogenicity of the endometrium is described as hyperechogenic, isoechogenic or hypoechogenic compared with the echogenicity of the myometrium^1^. Endometrial echogenicity should be defined as ‘uniform’ if the endometrium is homogeneous, with symmetrical anterior and posterior sides. A uniform endometrium generally has either a three‐layer pattern (as seen in the proliferative phase of the cycle) or a homogeneous hyperechogenic pattern (as seen in the late secretory phase of the cycle). Endometrial echogenicity is defined as ‘non‐uniform’ if the endometrium appears heterogeneous, asymmetrical or cystic.

##### Midline

**Figure 6:**
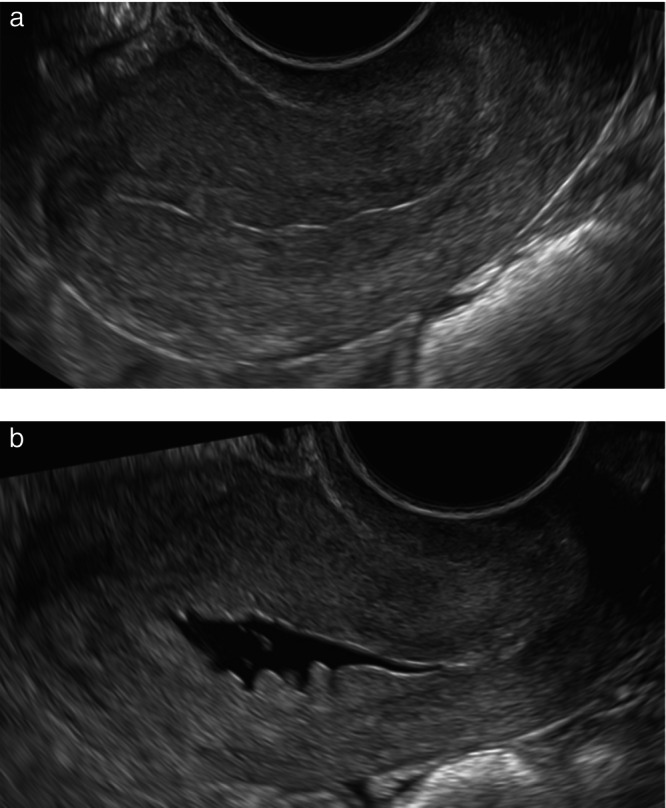
Ultrasonographic examples of a non‐linear endometrial midline, in sagittal section. (a) Without fluid instillation, it can be seen that the endometrial midline is not linear. (b) After fluid instillation, note the irregular and polypoid delineation of the cavity.

The endometrial midline is defined as ‘linear’ if a straight interface within the endometrium is visualized, as ‘non‐linear’ if a waved interface is seen (Figure 6, Videoclips S1 and S2), as ‘irregular’ or as ‘not defined’ in the absence of a distinct interface^1^.

##### Endometrial–myometrial junction

The endometrial–myometrial junction is the interface between the basal endometrium and the inner myometrium. It is described as ‘regular’, ‘irregular’, ‘interrupted’ or ‘not defined’^1^. The inner myometrium underlying the endometrium is also called the ‘junctional zone’.

### Clinical relevance: management of abnormal uterine bleeding

The aim of gynecological ultrasonography in women with abnormal bleeding is to exclude or detect intracavitary lesions such as endometrial polyps, intracavitary fibroids, endometrial hyperplasia, endometrial cancer or retained pregnancy tissue^61^, ^62^, ^63^.

**Figure 7:**
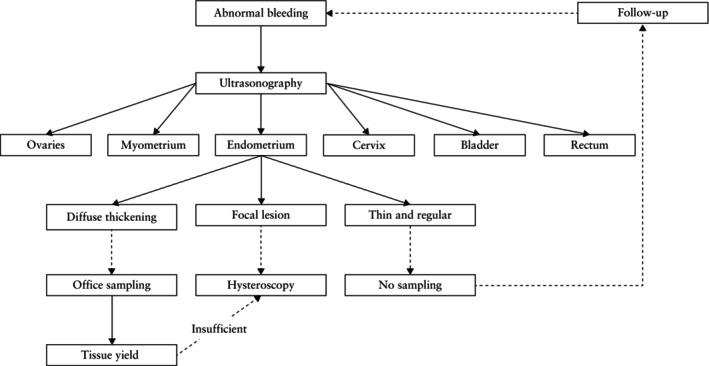
Diagnostic algorithm for women with abnormal uterine bleeding: stepwise diagnostic approach. An ultrasound examination is performed, first, to search for lesions in the bladder, rectum, ovaries, cervix or myometrium, and second, to evaluate the endometrium, triaging women in whom office sampling is most likely to yield an accurate histological diagnosis, those in whom hysteroscopy is indicated and those who do not need further testing.

Although the focus of this Consensus Statement is on the endometrium, when investigating abnormal uterine bleeding an ultrasound examination allows a broader evaluation of adjacent structures, including the myometrium (e.g. adenomyosis, fibroids, myometrial vascularity), ovaries (e.g. corpus luteum, endometrioma, granulosa cell tumor, tubo‐ovarian abscess), Fallopian tubes (e.g. hydrosalpinx, pyosalpinx, hematosalpinx, tubal pregnancy, tubal cancer), bladder (e.g. transitional‐cell carcinoma, endometriosis, lithiasis, cystitis), cervix (e.g. endocervical polyp, cervical carcinoma, cervical involvement of endometrial carcinoma) and rectum (e.g. endometriosis, malignancy) (Figure 7).

#### Differentiating between malignant and benign lesions

In women with abnormal uterine bleeding, endometrial malignancy is unlikely if the total endometrial thickness is considered ‘thin’^64^, ^65^, if the endometrium has a uniform three‐layer pattern or if the endometrial midline is regular and linear^4^, ^66^, ^67^. In women presenting with postmenopausal bleeding, the endometrium is considered ‘thin’ if its thickness is ≤ 4 mm, but the clinician may decide to use a lower threshold (e.g. 3 mm) to increase the sensitivity and negative predictive value (Table 2).

**Table 2:** Summary estimates of sensitivity, specificity, positive (PPV) and negative (NPV) predictive values and positive (LR+) and negative (LR−) likelihood ratios to detect endometrial cancer at different thresholds of endometrial thickness

Threshold	Sensitivity (%)	Specificity (%)	PPV	NPV	LR+	LR−
3 mm	96.2–100^59^, ^63^, ^64^, ^68^	23.0–42.1^59^, ^63^, ^64^, ^68^	7.3–9^59^, ^68^	99.7–100^59^, ^68^	1.29^59^	0^59^
4 mm	94.8–96.0^59^, ^63^, ^64^, ^68^	32.0–53.0^59^, ^63^, ^64^, ^68^	10.0–12.7^59^, ^68^	99.0–99.4^59^, ^68^	1.41^59^	0.13^59^
5 mm	88.0–96.2^59^, ^63^, ^64^, ^68^	40.0–54.0^59^, ^63^, ^64^, ^68^	11.0–21.1^59^, ^68^	98.0–99.3^59^, ^68^	1.47^59^	0.31^59^

Studies from which the summary estimates are derived are given.

**Figure 8:**
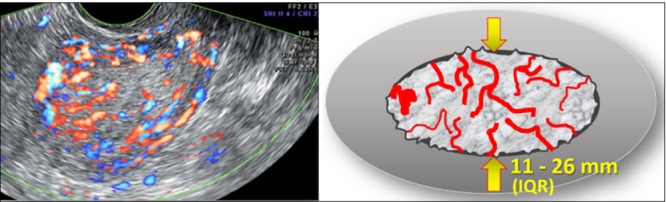
Color Doppler ultrasound image and diagram showing features suggestive of endometrial cancer^4^: thickened endometrium (arrows; interquartile range (IQR) = 11–26 mm in women with cancer), heterogeneous endometrium, presence of irregular cysts, non‐visible endometrial midline, irregular, interrupted or ill‐defined endometrial–myometrial junction, multiple vessels of focal or multifocal origin and/or high color score of 3–4. For postmenopausal women, an endometrial thickness > 4 mm is generally used to define thickened endometrium.

Alongside endometrial thickness, endometrial morphology, adequate visualization of the endometrial–myometrial junction and vascularization are also important in differentiating malignant from benign lesions. Endometrial cancer is typically associated with a thickened endometrium (i.e. > 4 mm in postmenopausal women), a heterogeneous endometrium, the presence of irregular cysts, a non‐visible endometrial midline, an irregular, interrupted or ill‐defined endometrial–myometrial junction, multiple vessels of focal or multifocal origin and/or a high color score of 3–4^4^, ^68^ (Figure 8).

#### Importance of menopausal status on endometrial thickness and other ultrasound features: the premenopausal endometrium

Endometrial thickness changes continuously in response to hormonal stimulation. Unlike after menopause, a cut‐off value for endometrial thickness is not useful prior to menopause, and endometrial features other than thickness are more relevant^4^. To investigate abnormal uterine bleeding, ultrasound examination is recommended in the first half of the menstrual cycle, after bleeding has stopped^1^, ^4^. In the secretory phase, the endometrium may appear thickened and more echogenic, and physiological endometrial changes may be difficult to distinguish from pathology. In such cases, a repeat ultrasound examination after the cessation of menstrual bleeding may be indicated.

##### Spontaneous cycle

In women with a spontaneous menstrual cycle, information about the day of the cycle and the presence or absence of a dominant follicle or corpus luteum will allow better interpretation of the endometrial features observed at ultrasound. Concordance or discordance between the endometrial appearance, the day of the cycle and the ovarian characteristics should be reported.

##### Hormonal therapy

Endometrial thickness is affected by the use and type of hormonal contraception (e.g. progestogen‐only pill, monophasic or sequential combined oral contraception). The endometrium of women on continuous hormonal therapy tends to be thin and more hyperechogenic.

##### Dealing with an IUCD or IUS in situ

In the presence of an IUCD/IUS, owing to shadowing, part of the underlying endometrium is hidden. The endometrium should be recorded as ‘not measurable’. The endometrium alongside the IUCD/IUS is generally visible and relevant sonographic features may be reported. In patients using a copper IUCD, the endometrium displays the usual cyclical hormonal changes, while it stays thin and hyperechogenic in women who have a levonorgestrel IUCD/IUS.

#### Importance of menopausal status on endometrial thickness and other ultrasound features: the postmenopausal endometrium

##### Without HRT

Without HRT, endometrial thickness is expected to be thin (i.e. ≤ 4 mm) after the menopause^4^.

##### With HRT (e.g. cyclical scheme, continuous scheme)

The endometrial thickness in women using HRT is affected by the HRT regimen, composition and dosage and varies according to the time during the pseudocycle at which the measurement is taken^69^. According to the British Menopause Society joint guidelines^70^, the cut‐off for a thickened endometrium is defined as 4 mm for women on continuous combined HRT (ccHRT) and 7 mm for women on sequential HRT (sHRT). However, there is insufficient evidence that endometrial thickness in women using ccHRT differs substantially from that in women using sHRT if the measurement is taken soon after the end of the planned withdrawal bleed. There has been even less research on the effect of current day ‘body‐identical’ HRT regimens on endometrial thickness. In the absence of robust data, clinicians should adopt the same conservative endometrial thickness cut‐off as that used for non‐HRT patients (i.e. 4 mm).

##### With selective estrogen receptor modulator (SERM) therapy

**Figure 9:**
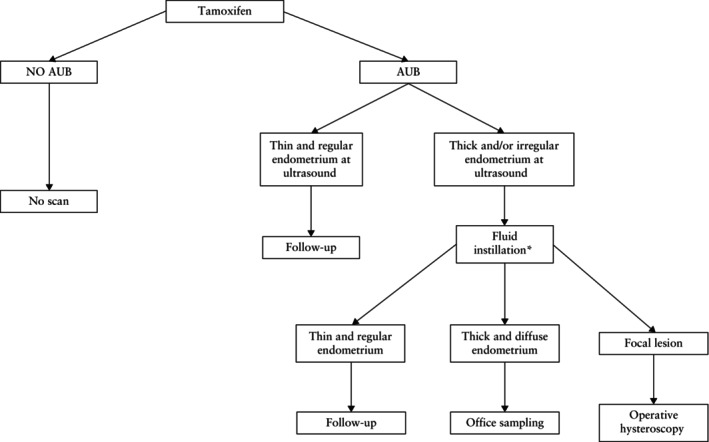
Diagnostic algorithm in patients taking tamoxifen. In those without abnormal uterine bleeding (AUB), ultrasonography is not indicated. In those presenting with AUB, ultrasonography will guide the clinician in triaging patients who do not need further testing (and may be followed up clinically), those in whom office sampling is most likely to yield an accurate histological diagnosis and those in whom hysteroscopy is indicated. *Saline‐contrast sonohysterography or gel‐instillation sonography.

Ultrasonography should be the first‐line examination for women with abnormal uterine bleeding on SERM therapy (e.g. tamoxifen)^71^. Cysts are frequently observed in women treated with tamoxifen. Cysts of various different sizes will typically be present in the subendometrial layer, within the (apparently) thickened endometrium or within glandulocystic polyps^72^. In the absence of abnormal uterine bleeding, there is no indication to perform a routine ultrasound evaluation of the endometrium in women on SERM therapy^71^, ^73^ (Figure 9).

### Incidental findings

Although there is no place for endometrial cancer screening in the general population^74^, ^75^, ^76^, gynecological ultrasonography is often performed in women without abnormal uterine bleeding for other indications, such as when there is suspicion of an ovarian cyst or pelvic pain^5^, ^77^.

Although concern regarding endometrial pathology may not have been the indication for the ultrasound scan, it remains a challenge to manage incidental endometrial ultrasound findings in the absence of abnormal uterine bleeding^77^. Many studies have focused on the risk of endometrial cancer in relation to endometrial thickness in the postmenopausal population^78^. However, endometrial thickness should not be the only sonographic feature that is taken into consideration when deciding on further invasive endometrial testing^79^.

#### Warning signs

**Figure 10:**
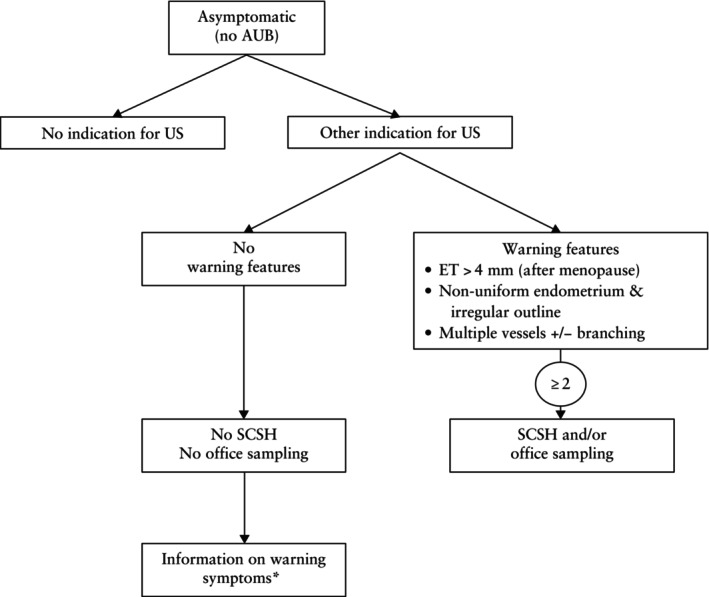
Diagnostic algorithm in women without abnormal uterine bleeding (AUB). In women without AUB, sonographic screening for endometrial pathology is not indicated. If an ultrasound scan (US) is performed for another indication, sonographic ‘alarm features’ within the endometrium (ET) are evaluated. These include: (1) a thickened ET > 4 mm in postmenopausal women, (2) non‐uniform endometrium with irregular outline and (3) multiple vessels with or without branching. In a woman without AUB, the incidental finding of two or more of these alarm features indicates that further testing should be considered. *The patient should be asked to return if she experiences further AUB. SCSH, saline contrast sonohysterography.

In a woman without abnormal uterine bleeding, further testing should be considered in the presence of two or more warning signs observed during ultrasound examination for another indication. Warning signs include: a thickened endometrium (> 4 mm in postmenopausal women); non‐uniform endometrium with an irregular outline; and multiple vessels with or without branching (Figure 10). However, this algorithm has yet to be validated in a randomized controlled trial. In the absence of robust evidence, the incidental finding of a thickened endometrium (> 4 mm in postmenopausal women) may prompt a more detailed examination during the scan, first to scrutinize both intrauterine and extrauterine features and second to actively exclude the other two warning signs. If both these steps are reassuring, it can be stated that no immediate further action is required. However, patients should be advised to remain vigilant for any postmenopausal bleeding that may occur subsequently, mandating further evaluation.

#### Intracavitary fluid

The presence of pre‐existing intracavitary fluid should be reported^1^, and the examiner should assess whether a possible explanation for the fluid can be found. Although anechoic intracavitary fluid after menopause due to endometrial atrophy is a common feature and does not deserve further investigation, fluid may also be secondary to bleeding due to intracavitary or tubal pathology (e.g. polyp, fibroid, hematometra with a thin endometrium in women taking contraception associated with intermenstrual spotting or cancer)^80^, ^81^. The echogenicity of intracavitary blood often has a ground‐glass appearance or may show a sedimentation level^1^.

#### Focal intracavitary endometrial lesions

**Figure 11:**
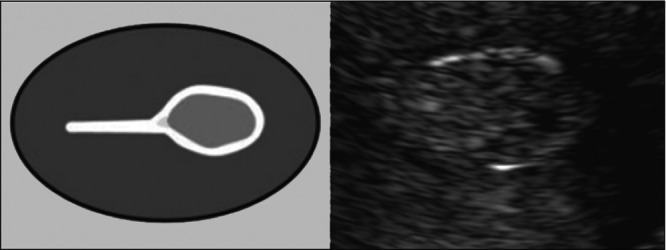
Diagram and ultrasound image showing the ‘bright‐edge’ sign (formed by the interface between the intracavitary lesion and the endometrium), suggestive of focal intracavitary endometrial pathology. Adapted from Leone *et al*.^1^.

Endometrial polyps are well‐circumscribed protrusions into the uterine cavity^73^, ^74^, ^75^. The presence of endometrial polyps can be suspected on grayscale ultrasound by the presence of a ‘bright edge’, seen as a sharp and smooth echogenic line that is generated by the interface between the polyp and the juxtaposed endometrium^1^, ^82^ (Figure 11).

**Figure 12:**
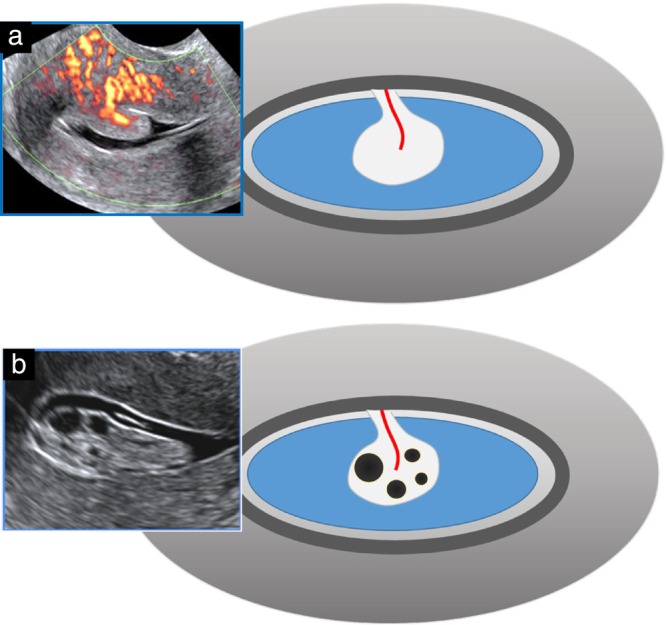
Power Doppler and grayscale ultrasound images and diagrams highlighting the differences between premenopausal (a) and postmenopausal (b) focal endometrial pathology on fluid‐instillation sonography. Before menopause, a polyp most often appears hyperechogenic and has a distinct dominant vessel, while after menopause, approximately half of polyps show regular internal cysts and the feeding vessel is often less distinct or even not visible. Adapted from Van den Bosch *et al*.^4^.

Endometrial cysts are commonly associated with endometrial polyps after menopause, but can also be seen in atrophic or hyperplastic endometria^4^, ^5^. The detection of polyps can be improved by dynamic assessment^13^. As with blood clots, targeted gentle pressure from the probe against the uterine corpus, or autonomic uterine peristalsis^28^, ^29^, ^30^, ^31^, can unmask the presence of intracavitary lesions by elongation or sliding (i.e. the ‘sliding (polyp) sign’)^1^. The use of color/power Doppler will typically demonstrate the presence of a dominant vessel originating from the myometrium and reaching into the central part of the endometrium (i.e. the ‘pedicle‐artery’ sign)^1^, ^4^, ^5^, ^26^. The risk of malignancy depends on various clinicodemographic factors^83^. Endometrial cancer within a polyp may be suspected on ultrasonography^3^, ^4^, ^5^, ^84^, ^85^ if the lesion has an irregular outline, if there are multiple internal vessels with an irregular branching pattern or if the lesion is large (> 18 mm)^3^, ^4^, ^5^, ^85^. The presence of intracavitary fluid (pre‐existing or after fluid instillation) improves visualization of the lesion's outline (Figure 12).

**Figure 13:**
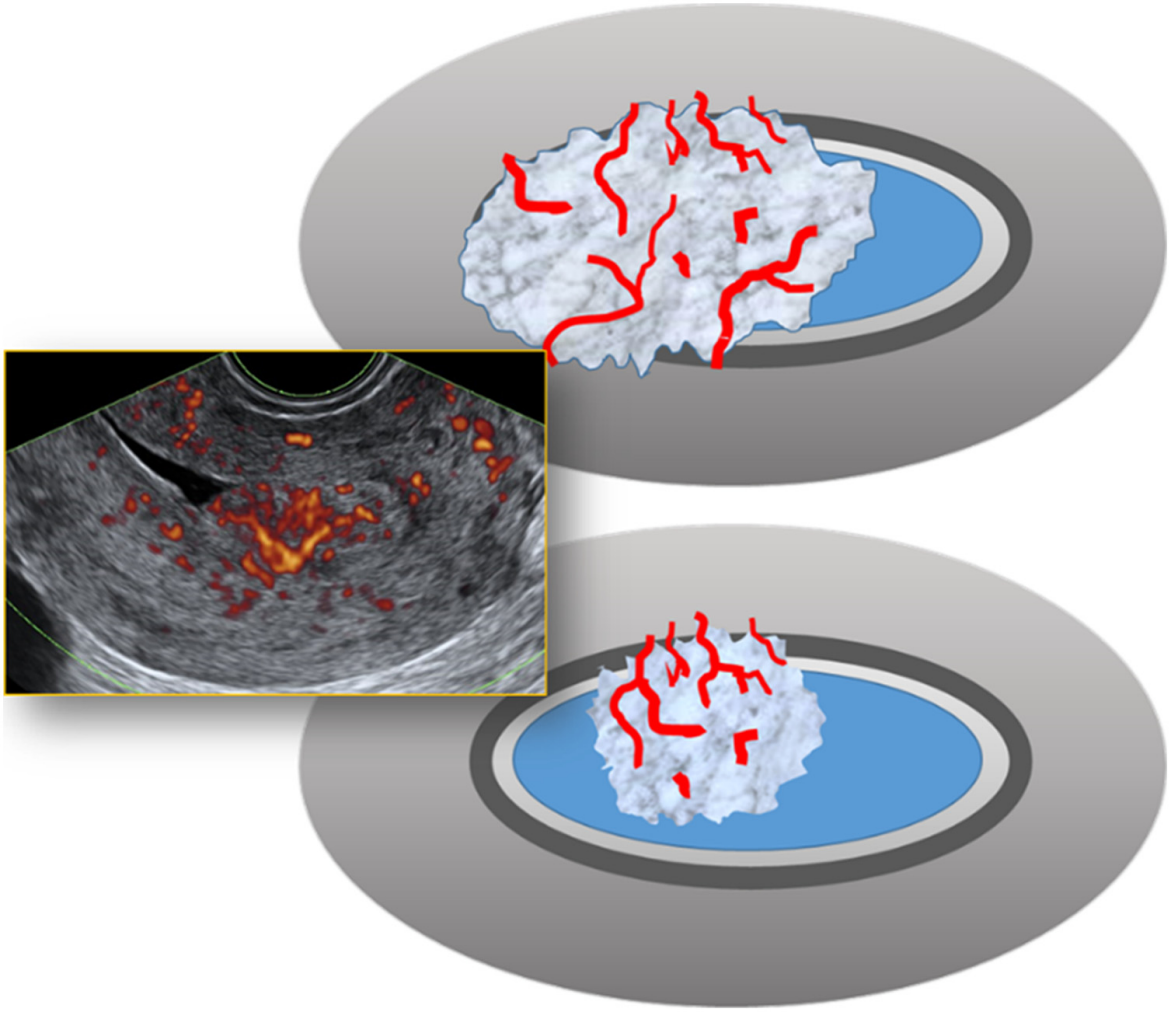
Power Doppler ultrasound image and diagrams showing multiple vessels with an irregular branching pattern, within an extended (upper diagram) or localized (lower diagram) intracavitary lesion with an irregular outline. Adapted from Van den Bosch *et al*.^4^.

**Figure 14:**
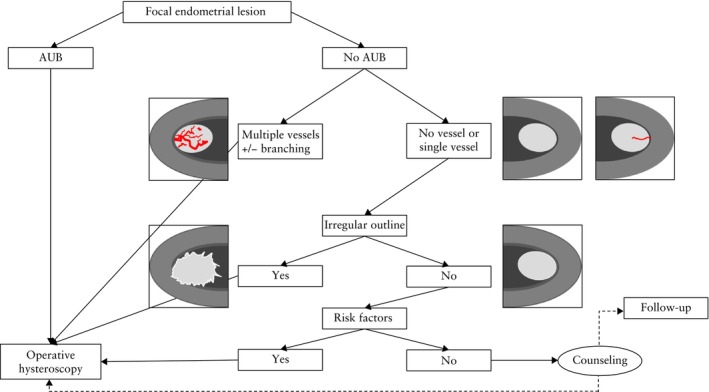
Diagnostic/management algorithm for focal endometrial lesions. The sonographic detection of a focal endometrial lesion (e.g. polyp) in a woman with abnormal uterine bleeding (AUB) warrants operative hysteroscopy with complete removal of the lesion. In the absence of AUB, a stepwise diagnostic approach is proposed. Hysteroscopy is indicated if multiple intralesional vessels, or an irregular lesion outline, are seen on ultrasound imaging. In the absence of abnormal sonographic features as well as of risk factors for endometrial malignancy, the patient may decide, after appropriate counseling, to undergo expectant management or hysteroscopy.

In women presenting with a focal endometrial lesion and abnormal uterine bleeding, hysteroscopic removal is recommended. For the more common clinical scenario of an incidentally identified endometrial polyp in asymptomatic premenopausal or postmenopausal patients, additional sonographic features can help to guide management. The presence of multiple vessels with or without an irregular branching pattern, as well as the observation of an irregular endometrial echogenicity or outline of the intracavitary lesion, should prompt in‐depth discussion on the need for histological diagnosis of the lesion (Figures 13 and 14). In the absence of such warning signs, the following management approaches may be considered: (1) expectant management with no immediate intervention; (2) interval reassessment to evaluate for spontaneous resolution at a later date; (3) sonohysterography to confirm that the lesion is solitary and well‐circumscribed prior to making a management decision.

(Socio)demographic characteristics^86^, clinical and sonographic findings^87^ and the patient's preference should be taken into account when considering further management.

##### Endometrial sampling

When focal lesions are found at sonohysterography, they should be removed using hysteroscopy, while when the endometrium is globally thickened blind sampling techniques may be adequate^88^.

If endometrial malignancy is suspected based on the sonographic features (Figures 7, 9 and 10), office endometrial sampling is indicated^89^ and should be processed with priority. Pipelle sampling has good accuracy to detect endometrial (pre)malignancy^89^, ^90^ and is a cost‐effective first diagnostic step^91^. Ultrasound guidance should be considered to optimize sampling adequacy if access to the uterine cavity is difficult. If the sample is inadequate or inconclusive or shows only benign findings, hysteroscopy is recommended.

During endometrial sampling, the tissue yield should be assessed and compared with the sonographic features. If there is minimal tissue yield in a woman with a thickened endometrium at ultrasound scan, a lesion has most probably been missed, and the results of the histology will most probably not be representative^92^, ^93^, ^94^, ^95^. In these cases, hysteroscopy with targeted biopsy is suggested as a diagnostic alternative (Figure 7)^96^. Office hysteroscopy is a cost‐effective alternative to immediate operative hysteroscopy^97^.

### Collaborators

**J. Preisler**, Departamento de Ginecología y Obstetricia, Facultad de Medicina, Clínica Alemana‐Universidad del Desarrollo, Santiago, Chile; Departamento de Ginecología y Obstetricia, Hospital Clínico Universidad de Chile, Santiago, Chile

**O. Rotenberg**, Department of Obstetrics & Gynecology and Women's Health, Albert Einstein College of Medicine/Montefiore Medical Center, Bronx, NY, USA

**H. Marret**, Department of Obstetrics, Gynecology and Reproductive Medicine, University Hospital of Tours, Tours, France

**G. Condous**, Acute Gynaecology, Early Pregnancy, and Advanced Endosurgery Unit, Nepean Hospital, Kingswood, NSW, Australia

**J. L. Alcázar**, Department of Obstetrics and Gynecology, Clinica Universidad de Navarra, School of Medicine, Pamplona, Spain

**D. Fischerová**, Department of Obstetrics and Gynecology, First Faculty of Medicine, Charles University and General University Hospital in Prague, Prague, Czech Republic

**U. Metzger**, Département d'Échographie en Gynécologie et Obstétrique, Centre d'Échographie de l'Odéon, Paris, France

**C. Van Pachterbeke**, Department of Obstetrics and Gynecology, Brugmann University Hospital, Brussels, Belgium

**D. Franchi**, Preventive Gynecology Unit, Division of Gynecology, European Institute of Oncology IRCCS, Milan, Italy

**M. Nisolle**, Department of Obstetrics and Gynecology, Hospital de La Citadelle, University of Liege, Liege, Belgium

**J. Huirne**, Department of Gynecology and Obstetrics, Research Institute ‘Reproduction and Development’, Amsterdam UMC, Location VUmc, Amsterdam, The Netherlands

**C. Fotopoulou**, Gynaecological Oncology, Department of Surgery and Cancer, Imperial College London, London, UK

**S. Goldstein**, Department of Obstetrics and Gynecology, New York University, New York City, NY, USA

## CITATION

This Consensus Statement should be cited as: ‘Van den Bosch T, Heremans R, Landolfo C, Epstein E, Leone FPG, Bourne T, Timmerman D and Collaborators. ISUOG Consensus Statement on sonographic assessment of the endometrium: how to perform a gynecological ultrasound scan and report the findings. *Ultrasound Obstet Gynecol*. 2026;67(2):241–258.

## Supporting information


**Videoclips S1 and S2** Grayscale (Videoclip S1) and power Doppler (Videoclip S2) ultrasonographic examples of a non‐linear midline.

## Data Availability

Data sharing is not applicable to this article as no new data were created or analyzed in this study.

## Appendix

**Appendix 1** Grades of recommendation and levels of evidence used in International Society of Ultrasound in Obstetrics and Gynecology Guidelines and Consensus Statements

**Table:**

Classification of evidence levels	
1++	High‐quality meta‐analyses, systematic reviews of randomized controlled trials or randomized controlled trials with low risk of bias
1+	Well‐conducted meta‐analyses, systematic reviews of randomized controlled trials or randomized controlled trials with low risk of bias
1–	Meta‐analyses, systematic reviews of randomized controlled trials or randomized controlled trials with high risk of bias
2++	High‐quality systematic reviews of case–control or cohort studies or high‐quality case–control or cohort studies with very low risk of confounding, bias or chance and high probability that the relationship is causal
2+	Well‐conducted case–control or cohort studies with low risk of confounding, bias or chance and moderate probability that the relationship is causal
2–	Case–control or cohort studies with high risk of confounding, bias or chance and significant risk that the relationship is not causal
3	Non‐analytical studies, e.g., case reports, case series
4	Expert opinion
*Grades of recommendation*	
A	At least one meta‐analysis, systematic review or randomized controlled trial rated as 1++ and applicable directly to the target population; or a systematic review of randomized controlled trials or a body of evidence consisting principally of studies rated as 1+ applicable directly to the target population and demonstrating overall consistency of results
B	Body of evidence including studies rated as 2++ applicable directly to the target population and demonstrating overall consistency of results; or extrapolated evidence from studies rated as 1++ or 1+
C	Body of evidence including studies rated as 2+ applicable directly to the target population and demonstrating overall consistency of results; or extrapolated evidence from studies rated as 2++
D	Evidence level 3 or 4; or evidence extrapolated from studies rated as 2+
Good practice point	Recommended best practice based on the clinical experience of the consensus group

## References

[1] Leone FP , Timmerman D , Bourne T , et al. Terms, definitions and measurements to describe the sonographic features of the endometrium and intrauterine lesions: a consensus opinion from the International Endometrial Tumor Analysis (IETA) group. Ultrasound Obstet Gynecol. 2010;35:103‐112. 20014360 10.1002/uog.7487

[2] Sladkevicius P , Installé A , Van Den Bosch T , et al. International Endometrial Tumor Analysis (IETA) terminology in women with postmenopausal bleeding and sonographic endometrial thickness ≥ 4.5 mm: agreement and reliability study. Ultrasound Obstet Gynecol. 2018;51:259‐268. 28715144 10.1002/uog.18813

[3] Epstein E , Fischerova D , Valentin L , et al. Ultrasound characteristics of endometrial cancer as defined by International Endometrial Tumor Analysis (IETA) consensus nomenclature: prospective multicenter study. Ultrasound Obstet Gynecol. 2018;51:818‐828. 28944985 10.1002/uog.18909

[4] Van den Bosch T , Verbakel JY , Valentin L , et al. Typical ultrasound features of various endometrial pathologies described using International Endometrial Tumor Analysis (IETA) terminology in women with abnormal uterine bleeding. Ultrasound Obstet Gynecol. 2021;57:164‐172. 32484286 10.1002/uog.22109

[5] Heremans R , Van Den Bosch T , Valentin L , et al. Ultrasound features of endometrial pathology in women without abnormal uterine bleeding: results from the International Endometrial Tumor Analysis study (IETA3). Ultrasound Obstet Gynecol. 2022;60:243‐255. 35385178 10.1002/uog.24910

[6] General Medical Council . Intimate examinations and chaperones. Accessed December 11, 2023.: https://www.gmc‐uk.org/ethical‐guidance/ethical‐guidance‐for‐doctors/intimate‐examinations‐and‐chaperones/intimate‐examinations‐and‐chaperones

[7] The British Medical Ultrasound Society . Transvaginal ultrasound examinations –guidance for practitioners. Accessed August 12, 2023. https://www.bmus.org/policies‐statements‐guidelines/professional‐guidance/guidance‐pages/tvguidance‐for‐practitioners/

[8] Torres A , Horodeńska M , Woźniakowska E , Borowik J . Anxiety connected with pelvic ultrasound in adolescents and their caregivers: comparison of transabdominal and transrectal approaches. J Pediatr Adolesc Gynecol. 2023;36:284‐290. 36764510 10.1016/j.jpag.2023.01.216

[9] Gentry‐Maharaj A , Sharma A , Burnell M , et al. Acceptance of transvaginal sonography by postmenopausal women participating in the United Kingdom Collaborative Trial of Ovarian Cancer Screening. Ultrasound Obstet Gynecol. 2013;41:73‐79. 22791597 10.1002/uog.12262

[10] Westerway SC , Basseal JM , Abramowicz J , Moran C , WFUMB Safety Committee . Recommendations for the cleaning of endocavity ultrasound transducers between patients. Ultrasound Med Biol. 2024;50:775‐778. 38485533 10.1016/j.ultrasmedbio.2024.02.011

[11] Knez J , Nardelli F , Van den Bosch T , Sladkevicius P , Valentin L , Jurkovic D . Imaging in gynecological disease (18): clinical and ultrasound characteristics of urinary bladder malignancies. Ultrasound Obstet Gynecol. 2020;56:453‐459. 31875325 10.1002/uog.21958

[12] Guerriero S , Condous G , van den Bosch T , et al. Systematic approach to sonographic evaluation of the pelvis in women with suspected endometriosis, including terms, definitions and measurements: a consensus opinion from the International Deep Endometriosis Analysis (IDEA) group. Ultrasound Obstet Gynecol. 2016;48:318‐332. 27349699 10.1002/uog.15955

[13] Testa AC , Van Holsbeke C , Mascilini F , Timmerman D . Dynamic and interactive gynecological ultrasound examination. Ultrasound Obstet Gynecol. 2009;34:225‐229. 19644933 10.1002/uog.7309

[14] Van den Bosch T , Dueholm M , Leone FP , et al. Terms, definitions and measurements to describe sonographic features of myometrium and uterine masses: a consensus opinion from the Morphological Uterus Sonographic Assessment (MUSA) group. Ultrasound Obstet Gynecol. 2015;46:284‐298. 25652685 10.1002/uog.14806

[15] Campbell S , Bhan V , Royston P , Whitehead MI , Collins WP . Transabdominal ultrasound screening for early ovarian cancer. Br Med J. 1989;299:1363‐1367. 2513964 10.1136/bmj.299.6712.1363 PMC1838264

[16] Hamed ST , Mansour SM . Surface transperineal ultrasound and vaginal abnormalities: applications and strengths. Br J Radiol. 2017;90:20170326. 28937267 10.1259/bjr.20170326 PMC6190777

[17] Teele RL , Share JC . Transperineal sonography in children. AJR. 1997;168:1263‐1267. 9129424 10.2214/ajr.168.5.9129424

[18] Timor‐Tritsch IE , Monteagudo A , Rebarber A , Goldstein SR , Tsymbal T . Transrectal scanning: an alternative when transvaginal scanning is not feasible. Ultrasound Obstet Gynecol. 2003;21:473‐479. 12768560 10.1002/uog.110

[19] Epstein E , Van Holsbeke C , Mascilini F , et al. Gray‐scale and color Doppler ultrasound characteristics of endometrial cancer in relation to stage, grade and tumor size. Ultrasound Obstet Gynecol. 2011;38:586‐593. 21547974 10.1002/uog.9038

[20] Eriksson LSE , Nastic D , Lindqvist PG , et al. Combination of Proactive Molecular Risk Classifier for Endometrial cancer (ProMisE) with sonographic and demographic characteristics in preoperative prediction of recurrence or progression of endometrial cancer. Ultrasound Obstet Gynecol. 2021;58:457‐468. 33314410 10.1002/uog.23573 PMC8457053

[21] Dueholm M , Hjorth IMD , Dahl K , Pedersen LK , Ørtoft G . Identification of endometrial cancers and atypical hyperplasia: development and validation of a simplified system for ultrasound scoring of endometrial pattern. Maturitas. 2019;123:15‐24. 31027672 10.1016/j.maturitas.2019.01.017

[22] Parsons AK , Lense JJ . Sonohysterography for endometrial abnormalities: preliminary results. J Clin Ultrasound. 1993;21:87‐95. 8381140 10.1002/jcu.1870210203

[23] Goldstein SR . Saline infusion sonohysterography. Clin Obstet Gynecol. 1996;39:248‐258. 8635304 10.1097/00003081-199603000-00023

[24] Exalto N , Stappers C , van Raamsdonk LAM , Emanuel MH . Gel instillation sonohysterography: first experience with a new technique. Fertil Steril. 2007;87:152‐155. 17097650 10.1016/j.fertnstert.2006.05.075

[25] Fleischer AC , Shappell HW , Parker LP , Hanemann CW . Color Doppler sonography of endometrial masses. J Ultrasound Med. 2002;21:861‐865. 12164571 10.7863/jum.2002.21.8.861

[26] Timmerman D , Verguts J , Konstantinovic ML , et al. The pedicle artery sign based on sonography with color Doppler imaging can replace second‐stage tests in women with abnormal vaginal bleeding. Ultrasound Obstet Gynecol. 2003;22:166‐171. 12905512 10.1002/uog.203

[27] Epstein E , Skoog L , Isberg PE , et al. An algorithm including results of gray‐scale and power Doppler ultrasound examination to predict endometrial malignancy in women with postmenopausal bleeding. Ultrasound Obstet Gynecol. 2002;20:370‐376. 12383320 10.1046/j.1469-0705.2002.00800.x

[28] Van den Bosch T , Van Schoubroeck D , Alcazar JL , Guerriero S , Martins WP . Importance of transient myometrial contractions in diagnosis of adenomyosis and congenital uterine anomalies. Ultrasound Obstet Gynecol. 2021;57:651‐653. 32250499 10.1002/uog.22036

[29] De Ziegler D , Brioschi PA , Fanchin R , Bulletti C , Ayoubi JM . Contractility of the non‐pregnant uterus. Infertil Reprod Med Clin North Am. 2003;14:309‐327.

[30] Abramowicz JS , Archer DF . Uterine endometrial peristalsis – a transvaginal ultrasound study. Fertil Steril. 1990;54:451‐454. 2204552

[31] Lyons EA , Taylor PJ , Zheng XH , Ballard G , Levi CS , Kredentser JV . Characterization of subendometrial myometrial contractions throughout the menstrual cycle in normal fertile women. Fertil Steril. 1991;55:771‐774. 2010002 10.1016/s0015-0282(16)54246-0

[32] de Kroon CD , de Bock GH , Dieben SW , Jansen FW . Saline contrast hysterosonography in abnormal uterine bleeding: a systematic review and meta‐analysis. BJOG. 2003;110:938‐947. 14550365 10.1111/j.1471-0528.2003.02472.x

[33] van Dongen H , de Kroon CD , Jacobi CE , Trimbos JB , Jansen FW . Diagnostic hysteroscopy in abnormal uterine bleeding: a systematic review and meta‐analysis. BJOG. 2007;114:664‐675. 17516956 10.1111/j.1471-0528.2007.01326.x

[34] Nieuwenhuis LL , Hermans FJ , Bij de Vaate AJM , et al. Three‐dimensional saline infusion sonography compared to two‐dimensional saline infusion sonography for the diagnosis of focal intracavitary lesions. Cochrane Database Syst Rev. 2017;5:CD011126. 28472862 10.1002/14651858.CD011126.pub2 PMC6481510

[35] Zuckerman J , Levine D , McNicholas MM , et al. Imaging of pelvic postpartum complications. AJR Am J Roentgenol. 1997;168:663‐638. 9057511 10.2214/ajr.168.3.9057511

[36] Van den Bosch T . Ultrasound in the diagnosis of endometrial and intracavitary pathology: an update. Australas J Ultrasound Med. 2012;15:7‐12. 28191132 10.1002/j.2205-0140.2012.tb00135.x PMC5025133

[37] Abu‐Zidan FM , Hefny AF , Corr P . Clinical ultrasound physics. J Emergencies, Trauma Shock. 2011;4:501‐503. 10.4103/0974-2700.86646 PMC3214508 22090745

[38] Lindheim SR , Morales AJ . Comparison of sonohysterography and hysteroscopy: lessons learned and avoiding pitfalls. J Am Assoc Gynecol Laparosc. 2002;9:223‐231. 11960054 10.1016/s1074-3804(05)60138-7

[39] Thijssen SG , Heremans RRG , Nderlita M , et al. Intrauterine fluid instillation and transtubal flow: a randomized controlled in vitro trial comparing gel and water. J Med Ultrasound. 2019;28:35‐40. 32368448 10.4103/JMU.JMU_29_19 PMC7194417

[40] Van den Bosch T , Verguts J , Daemen A , et al. Pain experienced during transvaginal ultrasound, saline contrast sonohysterography, hysteroscopy and office sampling: a comparative study. Ultrasound Obstet Gynecol. 2008;31:346‐351. 18307203 10.1002/uog.5263

[41] Unlu BS , Yilmazer M , Koken G , et al. Comparison of four different pain relief methods during hysterosalpingography: a randomized controlled study. Pain Res Manag. 2015;20:107‐111. 25848848 10.1155/2015/306248 PMC4391438

[42] Wolman I , Gordon D , Yaron Y , Kupferminc M , Lessing JB , Jaffa AJ . Transvaginal sonohysterography for the evaluation and treatment of retained products of conception. Gynecol Obstet Invest. 2000;50:73‐76. 10965186 10.1159/000010285

[43] American College of Obstetricians and Gynecologists' Committee on Gynecologic Practice . Technology Assessment No. 12: sonohysterography. Obstet Gynecol. 2016;128:e38‐e42. 27454735 10.1097/AOG.0000000000001588

[44] Egarter C , Krestan C , Kurz C . Abdominal dissemination of malignant cells with hysteroscopy. Gynecol Oncol. 1996;63:143‐144. 8898185 10.1006/gyno.1996.0294

[45] Arikan G , Reich O , Weiss U , et al. Are endometrial carcinoma cells disseminated at hysteroscopy functionally viable? Gynecol Oncol. 2001;83:221‐226. 11606075 10.1006/gyno.2001.6380

[46] Berry E , Lindheim SR , Connor JP , et al. Sonohysterography and endometrial cancer: incidence and functional viability of disseminated malignant cells. Am J Obstet Gynecol. 2008;199:240.e1‐240.e8. 10.1016/j.ajog.2008.03.042 18456240

[47] Bese T , Demirkiran F , Guralp O , Sanioglu C , Arvas M . Transtubal transport of carcinoma cells into the peritoneal cavity after saline infusion via transcervical route in patients with endometrial carcinoma. Int J Gynecol Cancer. 2009;19: 682‐685. 19509571 10.1111/IGC.0b013e3181a48c7f

[48] Alcázar JL , Errasti T , Zornoza A . Saline infusion sonohysterography in endometrial cancer: assessment of malignant cells dissemination risk. Acta Obstet Gynecol Scand. 2000;79:321‐322. 10746850

[49] Cosmi E , Saccardi C , Litta P , Nardelli GB , Dessole S . Transvaginal ultrasound and sonohysterography for assessment of postpartum residual trophoblastic tissue. Int J Gynaecol Obstet. 2010;110:262‐264. 20488441 10.1016/j.ijgo.2010.03.036

[50] Pfeifer SM , Attaran M , Goldstein J , et al. ASRM müllerian anomalies classification 2021. Fertil Steril. 2021;116:1238‐1252. 34756327 10.1016/j.fertnstert.2021.09.025

[51] Grimbizis GF , Di Spiezio SA , Saravelos SH , et al. The Thessaloniki ESHRE/ESGE consensus on diagnosis of female genital anomalies. Gynecol Surg. 2016;13:1‐16. 26918000 10.1007/s10397-015-0909-1 PMC4753246

[52] Ludwin A , Martins WP , Nastri CO , et al. Congenital Uterine Malformation by Experts (CUME): better criteria for distinguishing between normal/arcuate and septate uterus? Ultrasound Obstet Gynecol. 2018;51:101‐109. 29024135 10.1002/uog.18923

[53] Ludwin A , Coelho Neto MA , Ludwin I , et al. Congenital Uterine Malformation by Experts (CUME): diagnostic criteria for T‐shaped uterus. Ultrasound Obstet Gynecol. 2020;55:815‐829. 31432589 10.1002/uog.20845

[54] Bega G , Lev‐Toaff AS , O'Kane P , Becker E , Kurtz AB . Three‐dimensional ultrasonography in gynecology. J Ultrasound Med. 2003;22:1249‐1269. 14620897 10.7863/jum.2003.22.11.1249

[55] Jordans IPM , de Leeuw RA , Stegwee SI , et al. Sonographic examination of uterine niche in non‐pregnant women: a modified Delphi procedure. Ultrasound Obstet Gynecol. 2019;53:107‐115. 29536581 10.1002/uog.19049 PMC6590297

[56] Benacerraf BR , Shipp TD , Bromley B . Three‐dimensional ultrasound detection of abnormally located intrauterine contraceptive devices which are a source of pelvic pain and abnormal bleeding. Ultrasound Obstet Gynecol. 2009;34:110‐115. 19565532 10.1002/uog.6421

[57] Sakhel K , Sinkovskaya E , Horton S , Beydoun H , Chauhan SP , Abuhamad AZ . Orientation of the uterine fundus in reference to the longitudinal axis of the body: a 3‐dimensional sonographic study. J Ultrasound Med. 2014;33:323‐328. 24449736 10.7863/ultra.33.2.323

[58] Abuhamad AZ . Clinical implications of the echo enhancement artifact in volume sonography of the uterus. J Ultrasound Med. 2006;25:1431‐1435. 17060429 10.7863/jum.2006.25.11.1431

[59] Van den Bosch T , Ameye L , Van Schoubroeck D , Bourne T , Timmerman D . Intra‐cavitary uterine pathology in women with abnormal uterine bleeding: a prospective study of 1220 women. Facts Views Vis Obgyn. 2015;7:17‐24. 25897368 PMC4402439

[60] Goldstein SR , Khafaga A . Ability to successfully image endometrium on transvaginal ultrasound in asymptomatic postmenopausal women. Ultrasound Obstet Gynecol. 2021;58:625‐629. 33998081 10.1002/uog.23667

[61] Munro MG , Critchley HOD , Fraser IS , FIGO Menstrual Disorders Committee . The two FIGO systems for normal and abnormal uterine bleeding symptoms and classification of causes of abnormal uterine bleeding in the reproductive years: 2018 revisions. Int J Gynaecol Obstet. 2018;143:393‐408. 30198563 10.1002/ijgo.12666

[62] Jain V , Munro MG , Critchley HOD . Contemporary evaluation of women and girls with abnormal uterine bleeding: FIGO Systems 1 and 2. Int J Gynecol Obstet. 2023;162:29‐42. 10.1002/ijgo.14946 PMC10952771 37538019

[63] Smith‐Bindman R , Kerlikowske K , Feldstein VA , et al. Endovaginal ultrasound to exclude endometrial cancer and other endometrial abnormalities. JAMA. 1998;280:1510‐1517. 9809732 10.1001/jama.280.17.1510

[64] Timmermans A , Opmeer BC , Khan KS , et al. Endometrial thickness measurement for detecting endometrial cancer in women with postmenopausal bleeding: a systematic review and meta‐analysis. Obstet Gynecol. 2010;116:160‐167. 20567183 10.1097/AOG.0b013e3181e3e7e8

[65] Goldstein SR . Modern evaluation of the endometrium. Obstet Gynecol. 2010;116:168‐176. 20567184 10.1097/AOG.0b013e3181dfd557

[66] Rotenberg O , Doulaveris G , Fridman D , et al. Long‐term outcome of postmenopausal women with proliferative endometrium on endometrial sampling. Am J Obstet Gynecol. 2020;223:896.e1‐896.e7. 10.1016/j.ajog.2020.06.045 32640199

[67] Rotenberg O , Fridman D , Doulaveris G , et al. Long‐term outcome of postmenopausal women with non‐atypical endometrial hyperplasia on endometrial sampling. Ultrasound Obstet Gynecol. 2020;55:546‐551. 31389091 10.1002/uog.20421

[68] Long B , Clarke MA , Morillo ADM , Wentzensen N , Bakkum‐Gamez JN . Ultrasound detection of endometrial cancer in women with postmenopausal bleeding: systematic review and meta‐analysis. Gynecol Oncol. 2020;157:624‐633. 32008795 10.1016/j.ygyno.2020.01.032

[69] Van den Bosch T , Van Schoubroeck D , Ameye L , De Brabanter J , Van Huffel S , Timmerman D . Ultrasound assessment of endometrial thickness and endometrial polyps in women on hormonal replacement therapy. Am J Obstet Gynecol. 2003;188:1249‐1253. 12748493 10.1067/mob.2003.272

[70] British Menopause Society . BMS Joint Guideline Management of unscheduled bleeding on hormone replacement therapy (HRT). Prepared on behalf of the BMS, in partnership with the British Society of Gynaecological Endoscopy, British Gynaecological Cancer Society, Faculty of Sexual & Reproductive Healthcare, Getting It Right First Time (GIRFT), Royal College of General Practitioners and the Royal College of Obstetricians & Gynaecologists. Accessed April 21, 2024. https://thebms.org.uk/publications/bms‐joint‐guidelines/management‐of‐unscheduled‐bleeding‐on‐hormone‐replacement‐therapy‐hrt/ 10.1177/20533691241254413 38743767

[71] Timmerman D , Deprest J , Bourne T , Den Berghe IV , Collins WP , Vergote I . A randomized trial on the use of ultrasonography or office hysteroscopy for endometrial assessment in postmenopausal patients with breast cancer who were treated with tamoxifen. Am J Obstet Gynecol. 1998;179:62‐70. 9704766 10.1016/s0002-9378(98)70294-7

[72] Heremans R , Timmerman D , Van den Bosch T . Endometrial Polyp, Visual Encyclopedia of Ultrasound in Obstetrics and Gynecology. www.isuog.org, December 21st 2019

[73] Committee Opinion No. 601: tamoxifen and uterine cancer. Obstet Gynecol. 2014;123:1394‐1397. 24848920 10.1097/01.AOG.0000450757.18294.cf

[74] PDQ® Screening and Prevention Editorial Board . PDQ Endometrial Cancer Screening. Bethesda MD: National Cancer Institute. Updated November 4, 2023. Accessed May 6, 2023. https://www.cancer.gov/types/uterine/hp/endometrial‐screening‐pdq

[75] Gerber B , Krause A , Müller H , et al. Ultrasonographic detection of asymptomatic endometrial cancer in postmenopausal patients offers no prognostic advantage over symptomatic disease discovered by uterine bleeding. Eur J Cancer. 2001;37:64‐71. 11165131 10.1016/s0959-8049(00)00356-7

[76] Gemer O , Segev Y , Helpman L , et al. Is there a survival advantage in diagnosing endometrial cancer in asymptomatic postmenopausal patients? An Israeli Gynecology Oncology Group study. Am J Obstet Gynecol. 2018;219:181.e1‐181.e6. 10.1016/j.ajog.2018.05.013 29792852

[77] Smith‐Bindman R , Weiss E , Feldstein V . How thick is too thick? When endometrial thickness should prompt biopsy in postmenopausal women without vaginal bleeding. Ultrasound Obstet Gynecol. 2004;24:558‐565. 15386607 10.1002/uog.1704

[78] Vitale SG , Riemma G , Haimovich S , et al. Risk of endometrial cancer in asymptomatic postmenopausal women in relation to ultrasonographic endometrial thickness: systematic review and diagnostic test accuracy meta‐analysis. Am J Obstet Gynecol. 2023;228:22‐35.e2. 35932873 10.1016/j.ajog.2022.07.043

[79] Heremans R , Guerriero S , Van den Bosch T . Risk of endometrial cancer in asymptomatic postmenopausal women in relation to ultrasonographic endometrial thickness. Am J Obstet Gynecol. 2023;229:85‐86. 10.1016/j.ajog.2023.03.012 36933692

[80] Goldstein SR . Postmenopausal endometrial fluid collections revisited: look at the doughnut rather than the hole. Obstet Gynecol. 1994;83:738‐740. 8164935

[81] Epstein E , Valentin L . Gray‐scale ultrasound morphology in the presence or absence of intrauterine fluid and vascularity as assessed by color Doppler for discrimination between benign and malignant endometrium in women with postmenopausal bleeding. Ultrasound Obstet Gynecol. 2006;28:89‐95. 16741893 10.1002/uog.2782

[82] Caspi B , Appelman Z , Goldchmit R , Ashkenazi M , Haruvy Y , Hagay Z . The bright edge of the endometrial polyp. Ultrasound Obstet Gynecol. 2000;15:327‐330. 10895454 10.1046/j.1469-0705.2000.00096.x

[83] Uglietti A , Buggio L , Farella M , et al. The risk of malignancy in uterine polyps: a systematic review and meta‐analysis. Eur J Obstet Gynecol Reprod Biol. 2019;237:48‐56. 31009859 10.1016/j.ejogrb.2019.04.009

[84] Hulka CA , Hall DA , McCarthy K , Simeone JF . Endometrial polyps, hyperplasia and carcinoma in postmenopausal women: differentiation with endovaginal sonography. Radiology. 1994;191:755‐758. 8184058 10.1148/radiology.191.3.8184058

[85] Ferrazzi E , Zupi E , Leone FP , et al. How often are endometrial polyps malignant in asymptomatic postmenopausal women? A multicenter study. Am J Obstet Gynecol. 2009;200(235):e1‐e6. 10.1016/j.ajog.2008.09.876 19027096

[86] Verbakel JY , Heremans R , Wynants L , et al. Risk assessment for endometrial cancer in women with abnormal vaginal bleeding: results from the prospective IETA‐1 cohort study. Int J Gynaecol Obstet. 2022;159:103‐110. 35044676 10.1002/ijgo.14097 PMC9546126

[87] Wynants L , Verbakel JYJ , Valentin L , et al. The risk of endometrial malignancy and other endometrial pathology in women with abnormal uterine bleeding: an ultrasound‐based model development study by the IETA group. Gynecol Obstet Invest. 2022;87:54‐61. 35152217 10.1159/000522524

[88] Epstein E , Ramirez A , Skoog L , Valentin L . Dilatation and curettage fails to detect most focal lesions in the uterine cavity in women with postmenopausal bleeding. Acta Obstet Gynecol Scand. 2001;80:1131‐1136. 11846711 10.1034/j.1600-0412.2001.801210.x

[89] Dijkhuizen FP , Mol BW , Brölmann HA , Heintz AP . The accuracy of endometrial sampling in the diagnosis of patients with endometrial carcinoma and hyperplasia: a meta‐analysis. Cancer. 2000;89:1765‐1772. 11042572

[90] Clark TJ , Mann CH , Shah N , Khan KS , Song F , Gupta JK . Accuracy of outpatient endometrial biopsy in the diagnosis of endometrial cancer: a systematic quantitative review. BJOG. 2002;109:313‐321. 11950187 10.1111/j.1471-0528.2002.01088.x

[91] Clark TJ , Barton PM , Coomarasamy A , Gupta JK , Khan KS . Investigating postmenopausal bleeding for endometrial cancer: cost‐effectiveness of initial diagnostic strategies. BJOG. 2006;113:502‐510. 16637894 10.1111/j.1471-0528.2006.00914.x

[92] Van den Bosch T , Van Schoubroeck D , Van Calster B , Cornelis A , Timmerman D . Pre‐sampling ultrasound evaluation and assessment of the tissue yield during sampling improves the diagnostic reliability of office endometrial biopsy. J Obstet Gynaecol. 2012;32:173‐176. 22296431 10.3109/01443615.2011.635223

[93] Leone FPG , Carsana L , Lanzani C , Vago G , Ferrazzi E . Sonohysterographic endometrial sampling and hysteroscopic endometrial biopsy: a comparative study. Ultrasound Obstet Gynecol. 2007;29:443‐448. 17390311 10.1002/uog.3981

[94] Moschos E , Ashfaq R , McIntire DD , Liriano B , Twickler DM . Saline‐infusion sonography endometrial sampling compared with endometrial biopsy in diagnosing endometrial pathology. Obstet Gynecol. 2009;113:881‐887. 19305334 10.1097/AOG.0b013e31819b3fc7

[95] Rotenberg O , Renz M , Reimers L , et al. Simultaneous endometrial aspiration and sonohysterography for the evaluation of endometrial pathology in women aged 50 years and older. Obstet Gynecol. 2015;125:414‐423. 25568988 10.1097/AOG.0000000000000631

[96] Clark TJ , Voit D , Gupta JK , Hyde C , Song F , Khan KS . Accuracy of hysteroscopy in the diagnosis of endometrial cancer and hyperplasia: a systematic quantitative review. JAMA. 2002;288:1610‐1621. 12350192 10.1001/jama.288.13.1610

[97] Moawad NS , Santamaria E , Johnson M , Shuster J . Cost‐effectiveness of office hysteroscopy for abnormal uterine bleeding. J Soc Laparoendosc Surg. 2014;18:1‐5. 10.4293/JSLS.2014.00393 PMC4154435 25392645

